# Antibiotic and healthcare exposure impact on dynamics of third-generation cephalosporin-resistant Enterobacterales colonisation in Cambodian children: a six-month cohort study

**DOI:** 10.1186/s13756-026-01754-3

**Published:** 2026-04-22

**Authors:** Cristina Ardura-Garcia, Sambou Bran, Poda Sar, Keang Suy, Sreymom Pol, Sue J Lee, Clare Louise Ling, Jukka Corander, Paul Turner

**Affiliations:** 1 Cambodia Oxford Medical Research Unit, Angkor Hospital for Children, Siem Reap, Cambodia; 2 Department of Paediatrics at the Malaga Mother-and‐Child Hospital, Hospital Regional Universitario de Malaga, Malaga, Spain; 3 Instituto de Investigacion Biomedica de Malaga (Biomedical Research Institute of Malaga), Malaga, Spain; 4 Faculty of Medicine, University of Puthisastra, Phnom Penh, Cambodia; 5 Mahidol-Oxford Tropical Medicine Research Unit, Faculty of Tropical Medicine, Mahidol University, Bangkok, Thailand; 6 Department of Infectious Diseases, Alfred Hospital and School of Translational Medicine, Monash University, Melbourne, Australia; 7 Centre for Tropical Medicine & Global Health, Nuffield Department of Medicine, University of Oxford, Oxford, UK; 8 Department of Biostatistics, University of Oslo, Oslo, Norway; 9 Parasites and Microbes, Wellcome Sanger Institute, Hinxton, UK; 10 Department of Mathematics and Statistics, University of Helsinki, Helsinki, Finland

**Keywords:** Antimicrobial resistance, Colonisation, Cambodia, Paediatrics, *Escherichia coli*, *Klebsiella pneumoniae*

## Introduction

Antimicrobial resistance (AMR) is one of the major current global public health threats and a leading cause of death worldwide [1]. The gastrointestinal tract (GI) serves as a key reservoir for potentially pathogenic and AMR bacteria, and colonisation with resistant organisms increases the risk of subsequent AMR infection [2]. Although AMR is a worldwide problem, its burden is highest in low- and middle-income countries (LMICs) [1], with South-East Asia considered the region at greatest risk for emergence and spread of AMR [3]. In Cambodia, high colonisation prevalence of extended-spectrum beta-lactamase producing Enterobacterales (ESBL-E) has been documented in children: 55% combined *Escherichia coli / Klebsiella pneumoniae* in older children presenting to healthcare [4]; 92% *E. coli* and 36% *K. pneumoniae* in children post-discharge [5].

Hospitalisation and broad-spectrum antibiotic use are the main determinants for acquisition of AMR *E. coli* and *K. pneumoniae* [6–8]. Travel to high prevalence AMR areas, chronic diseases, previous surgeries, invasive procedures, dietary habits and intrafamilial transmission have also been identified as risk factors for AMR bacterial colonisation [6, 9, 10]. However, risk factors may vary by region, especially in LMICs. In Cambodian children, hospital admission and intestinal parasites were identified as independent risk factors for AMR *E. coli* and *K. pneumoniae* colonisation [4]. On the other hand, little is known about the long-term temporal dynamics of carriage after exposure, especially in children. Most data comes from studies on adults returning to Europe after international travel, which have shown a high proportion of gut colonisation with multidrug-resistant Enterobacterales (24–51%), especially when returning from India (72% in a meta-analysis) [11–15]. Persistence of colonisation occurred in 36% of the colonised travellers 1 month later [12], and in 5–28% 6 months later [11, 14, 15], associated with a vegetarian diet, travel to Asia, high relative abundance of AMR Enterobacterales, gut microbiota composition and cat ownership [11, 12, 14, 15]. However, it is unclear whether we may generalise these findings to Cambodian children. Although genotypically-verified data are relatively sparse, GI duration of colonisation by AMR Enterobacterales in children may be prolonged [16–19].

Understanding the temporal dynamics of AMR colonisation is crucial for designing future strategies to reduce its burden. Details of the long-term temporal dynamics of AMR bacterial colonisation and the factors that drive them remain relatively scarce in high disease burden LMIC populations, where effective interventions to prevent infections are most needed. We aimed to define the proportion of Cambodian children under 5 years old who were colonised by AMR Enterobacterales on presentation for healthcare, identify risk factors for colonisation at presentation, describe the colonisation dynamics over time, and the roles of healthcare and antibiotic exposure for the gain or loss of colonisation over time.

## Methods

### Study design, setting and ethics

COMRU-META was a prospective clinical cohort study of children seen at Angkor Hospital for Children (AHC), a non-governmental paediatric referral hospital based in Siem Reap, Cambodia. Cambodia is a lower-middle-income country [20], with an approximate under-five population of 1.8 million children in 2024 [16]. Siem Reap province in northwestern Cambodia has a population of around 1 million people (2019), predominantly rural despite its capital being a major cultural and tourism hub, and with age structure similar to national patterns of a young demographic [17]. AHC has 73 inpatient beds and provides free primary-to-tertiary care to children under 16 years, with ~ 100,000 outpatient visits and 5500 admissions annually, irrespective of geographic location.

The study was approved by the AHC Executive Committee (reference 0102 − 21 AHC), Cambodia National Ethics Committee for Health Research (NECHR, references 079-NECHR (23/04/2021) / 090-NECHR (18/04/2022) / 089-NECHR (17/03/2023)), and Oxford Tropical Research Ethics Committee (OxTREC, reference 514 − 21). Findings were reported in line with Strengthening the Reporting of Observational Studies in Epidemiology (STROBE) [18] and the Microbiology Investigation Criteria for Reporting Objectively (MICRO) [19] guidelines.

### Participants

We included children 1–59 months old visiting the outpatient (OPD) and inpatient (IPD) departments for non-elective healthcare during 2021–2022, whose parents or legal representatives consented to the child’s participation. Exclusion criteria were: residing outside Siem Reap district, systemic antibiotic use for current illness (to avoid culture suppression and bacterial selection of current colonisation), chronic medical condition (immunosuppression, active chronic infections, active cardiorespiratory conditions, or upper respiratory tract or intestinal tract abnormalities) or AHC prescribed antibiotic started more than 1 h before enrolment.

### Study procedures

Baseline clinical, treatment, and environmental data were collected at enrolment along with nasopharyngeal and rectal swabs (RS), either prior to departure from the OPD or on the day of IPD admission, though only RS were used for this analysis. Hospital admission and antibiotic treatment data were obtained from medical records and the AHC hospital information system. Household and environmental data were captured via administration of a questionnaire to the participant’s parent/legal representative. Children were followed up for 6 months with clinical visits and RS at 1, 3 and 6 months and telephone follow-up at 2, 4 and 5 months. At each follow-up a short questionnaire on time-varying exposures was completed (acute infections, healthcare and antibiotic exposure).

### Sample handling

RS were cultured onto selective chromogenic agar plates and incubated aerobically at 37 °C for 24 h to detect colonisation by 3^rd^-generation cephalosporin-resistant Enterobacterales (3GC-R-E) and carbapenem resistant Enterobacterales (CRE) *E. coli* (Ec) and *K. pneumoniae* (Kpn) (CHROMagar ESBL and KPC media). Target species were identified by MALDI-TOF mass-spectrometry (bioMerieux VITEK MS; Knowledge Base V3.2.0) and standard microbiological techniques. Antimicrobial susceptibilities were determined by VITEK 2 (bioMerieux) testing using AST-GN84 cards that included ESBL detection, and interpreted using 2022 Clinical and Laboratory Standard Institute (CLSI) Antimicrobial Susceptibility Testing guidelines [21]. Multidrug-resistance (MDR) was defined as non-susceptibility to ≥ 1 agent in ≥ 3 antimicrobial classes tested, excluding intrinsic resistance [21].

### Definitions of risk factors and outcomes

Potential risk factors for colonisation with AMR bacteria were selected based on previous literature. These included data from the questionnaires: sociodemographic information (age, sex), environmental exposures (pets and farm animals, number of people in the household, access to a toilet inside the house and handwashing basin within 2 m of the toilet, school or day care attendance), current breastfeeding, comorbidities, healthcare exposure in the previous 3 months (any healthcare consultation and hospital admission), and antibiotic use in the previous 4 weeks (shorter period to avoid recall bias). No specific information on diagnosis of previous healthcare contacts were collected, as these may have taken place outside of AHC and parent/legal representative recall may have been unreliable. Also included were weight and height to estimate body mass index (BMI), that were transformed into Z-scores using World Health Organisation references values [22]. For the Cox regression analysis, antibiotic and healthcare exposure extracted from follow-up visit forms were defined as ‘since previous visit’. Outcomes of interest were the presence of 3GC-R and CRE Ec and Kpn in RS, and the time-to-gain or -loss of 3GC-R Ec and Kpn colonisation. ‘3GC-R’ was defined as isolates that were either ESBL-positive or resistant to 3rd generation cephalosporins in the VITEK2 susceptibility testing. A ‘gain’ episode was defined as a visit when a child non-colonised in the previous visit became colonised. A ‘loss’ episode was defined as a visit when a child colonised in the previous visit became non-colonised.

### Statistical analyses

Demographic, clinical, treatment and colonisation data were summarised using descriptive statistics. GI colonisation proportions by AMR bacteria were estimated at each time point and dynamics shown with Sankey diagrams. Risk factors for 3GC-R and CRE Ec and Kpn colonisation at baseline were identified by univariable and multivariable logistic regression models (described further in the Supplementary Materials). Kaplan Meier curves were used to represent time-to-gain and time-to-loss of 3GC-R Ec and Kpn colonisation. Cox regression analysis was performed to study the effect of healthcare (any or inpatient) and antibiotic (any or 3rd generation cephalosporin) on the time-to-gain and time-to-loss of 3GC-R Ec and Kpn colonisation. For colonisation gain or loss, time to the event was defined as the mid-point between the two time points at which colonisation differed (from non-colonised to colonised or vice versa). Periods with no gain or loss were censored at the time of the last visit. Healthcare and antibiotic exposure were included as time-varying covariates. These models were adjusted for sex, age and relevant risk factors identified at baseline (toilet inside the house, available hand washing basin, weight-for-age Z score and current breastfeeding). Children with missing values for included variables were excluded from the analyses. No imputation for missing values was attempted. Estimates of effect (odds ratios, OR and hazard ratios, HR) are reported together with 95% confidence intervals (CI). The R statistical programme [23] version 4.3.0 was used for computation.

## Results

**Fig. 1:**
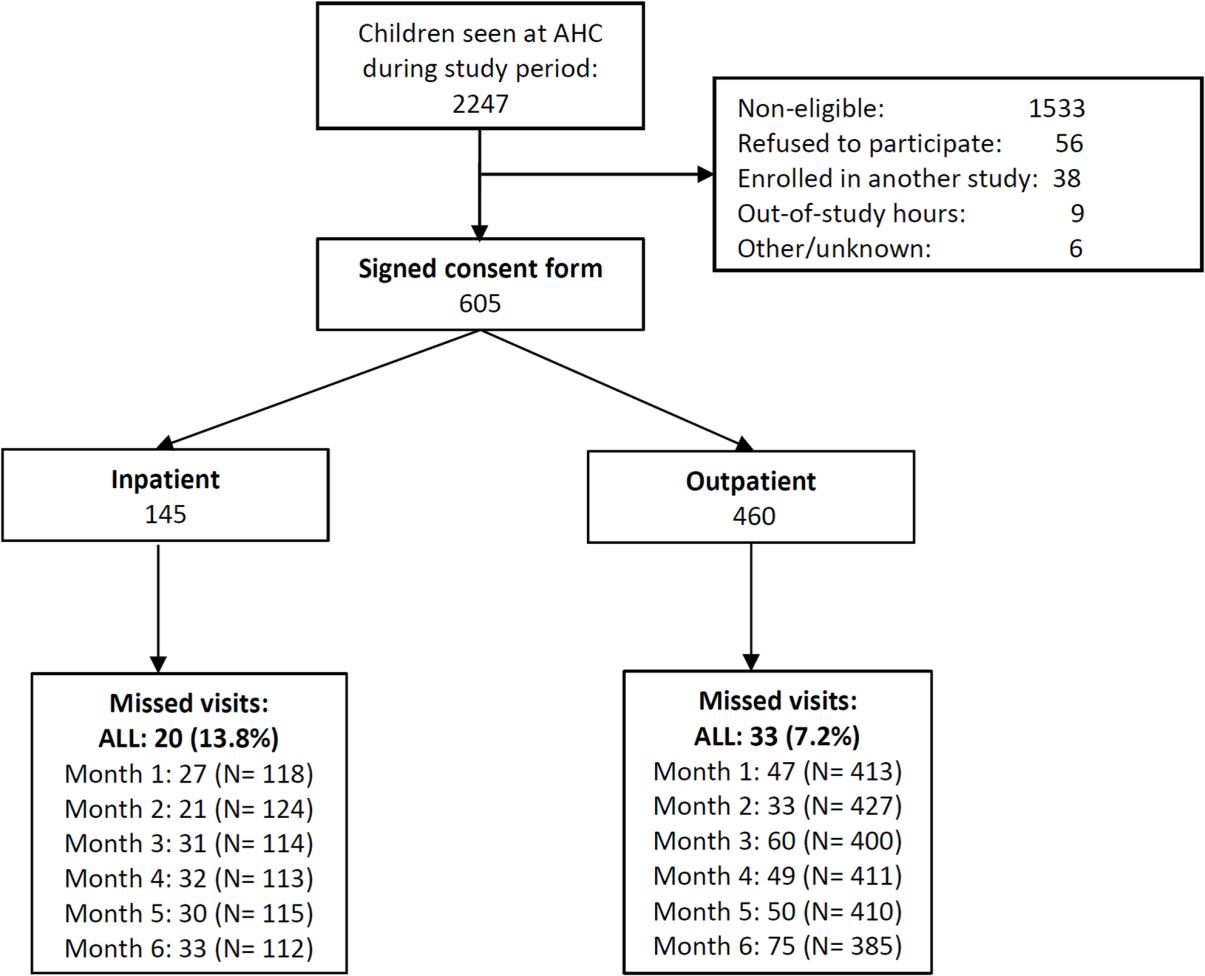
Study population flow. AHC: Angkor hospital for children. N= number of children attending each visit

During the study period, 661 children were eligible and invited to participate, of whom 605 (92%) legal representatives agreed to participate (Fig. 1). The study cohort consisted of 460 outpatients and 145 inpatients. The study population characteristics at enrolment are documented in Table 1 and Supplementary Table 1. Median age was 1.4 years (interquartile range, IQR, 0.8–2.4 years), 47% were female, and few (21, 4%) attended day-care or school. Mean BMI and weight-for-age Z-scores were less than 0 for outpatients, and less than − 1 among those admitted to hospital. One third (202, 33%, mostly < 1-year olds) were currently breastfeeding. Most children (511, 85%) reported recent healthcare contact and 41 (7%) reported antibiotic intake in the last month. Common diagnoses were upper respiratory infection (258, 43%) and gastroenteritis (115, 19%) (Supplementary Table 1). Among outpatients, 50/460 (11%) were prescribed an antibiotic, and among inpatients 17/145 (12%) were treated empirically with ceftriaxone or cefotaxime and 1 (0.7%) with meropenem.

**Table 1:** Characteristics at enrolment of children under 5 years old presenting at the Angkor Hospital for Children for healthcare during 2021 to 2022 stratified by those who were lost-to-follow-up and those who attended at least one visit

Sociodemographics:	*N*	All (*N* = 605)	Non-LTFU (*N* = 552)	LTFU (*N* = 53)	*p*- value
Female sex	605	285 (47)	264 (48)	21 (40)	0.3
Age (median, IQR) (years)	605	1.4 (0.8–2.4)	1.4 (0.8–2.4)	1.3 (0.6–2.4)	0.4
Environment					
People in hh (median, IQR)	605	5 (4–7)	5 (4–7)	5 (4–6)	0.6
Any animal in household	605	376 (62)	343 (62)	33 (62)	> 0.9
Pets (dogs/cats)	605	298 (49)	206 (37)	19 (36)	0.8
Farm animals	605	225 (37)	275 (50)	23 (43)	0.4
Toilet in house					
Inside	598	334 (56)	306 (55)	28 (53)	0.7
With basin for handwashing	605	494 (83)	449 (81)	45 (85)	0.5
School/day care attendance	605	21 (4)	17 (3.1)	4 (7.5)	0.10
Personal history					
BMI Z score (mean, SD)	538	-0.66 (1.19)	-0.65 (1.18)	-0.74 (1.27)	0.8
Weight for age Z score (mean, SD)	605	-0.93 (1.32)	-0.95 (1.31)	-0.77 (1.51)	0.2
Breastfeeding currently	605	202 (33)	187 (34)	15 (28)	0.4
Comorbidities	605	15 (2)	15 (2.7)	0 (0)	0.6
Any health care previous 3 months	605	511 (85)	469 (85)	42 (79)	0.3
Inpatient	604	58 (10)	54 (9.8)	4 (7.5)	0.6
Surgery	602	3 (0.5)	3 (0.5)	0 (0)	> 0.9
Healthcare visit	605	352 (58)	323 (59)	29 (55)	0.6
Pharmacy consultation	605	318 (53)	290 (53)	28 (53)	> 0.9
Traditional healer	604	19 (3)	14 (3)	5 (9)	0.019
Antibiotics previous 4 weeks	549	41 (7)	38 (7)	3 (6)	> 0.9
Amoxicillin (% of abx ‘yes’)		8 (20)	8 (21)	0 (0)	
Ceftriaxone / cefotaxime		4 (9)	4 (11)	0 (0)	
Amoxicillin-clavulanate		1 (2)	1 (3)	0 (0)	
Ofloxacin		1 (2)	1 (3)	0 (0)	
Metronidazole		1 (2)	1 (3)	0 (0)	
Meropenem		1 (2)	0 (0)	1 (33)	
Unknown		25 (61)	23 (61)	2 (67)	
Colonisation status	605				
3GC-R *E. coli*		512 (85)	466 (84)	46 (87)	0.6
CRE *E. coli*		13 (2)	11 (2.0)	2 (3.8)	0.3
3GC-R *K. pneumoniae*		162 (27)	146 (26)	16 (30)	0.6
CRE *K. pneumoniae*		8 (1)	6 (1.1)	2 (3.8)	0.15

Abx: antibiotics; BMI: body mass index; IQR: interquartile range; LTFU: lost to follow-up (missed all the follow-up visits); SD: standard deviation

### Bacterial colonisation and antimicrobial resistance at enrolment

From 605 baseline RS, 531 (88%) children were colonised with a 3GC-R-E (512 Ec, 162 Kpn, 143 both) and 21 (3%) by CRE (13 Ec, 8 Kpn) at enrolment (Table 1). Four and three children were colonised, respectively, with both an 3GC-R and a CRE Ec or Kpn. 3GC-R and CRE-Kpn colonisation decreased with age, being most prevalent in < 1-year olds admitted to hospital (Supplementary Fig. 1), whilst 3GC-R-Ec colonisation peaked in 1-year olds and CRE-Ec carriage increased with age. Of the 525 isolated Ec at enrolment, 129 (25%) were ampicillin-gentamicin-resistant, 232 (44%) fluoroquinolone-resistant, and 359 (68%) were MDR. The respective results for the 170 isolated Kpn were: 37 (21%), 96 (55%) and 106 (61%) (Supplementary Table 2). AMR data are summarised in Supplementary Table 2.

### Risk factors for AMR bacterial colonisation at enrolment

**Fig. 2:**
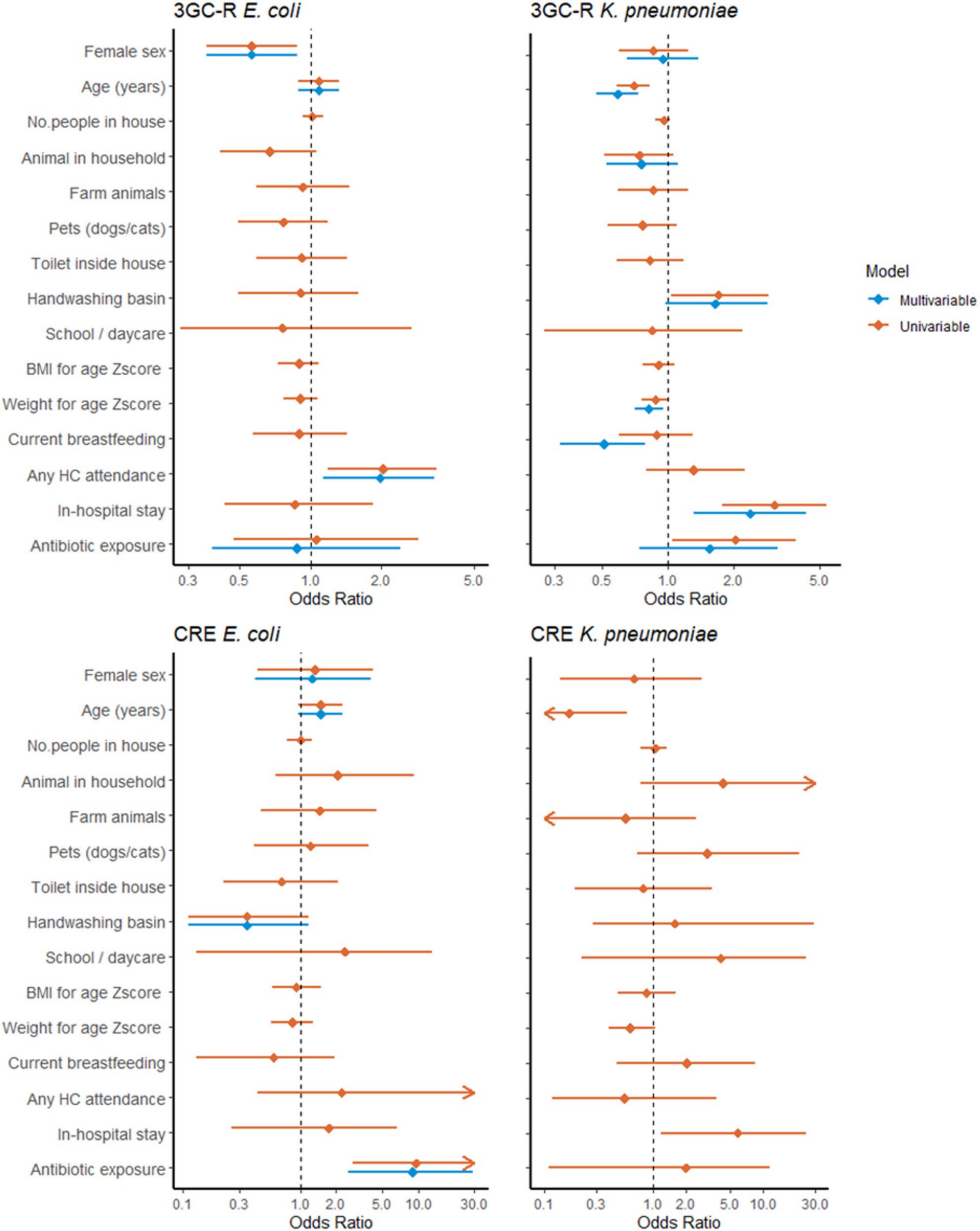
Association between sociodemographic, personal history and environmental factors with gastrointestinal colonisation of 3rd-generation cephalosporin resistant (3GC-R) and carbapenem-resistant (CRE) *Escherichia coli* and *Klebsiella pneumoniae* in children under 5 years old seen at Angkor Hospital for Children, at enrolment. *N* = 605

Independent risk factors for 3GC-R-Ec colonisation at enrolment were male sex (Adjusted OR, AOR, 0.56 for female sex, 95% CI 0.36–0.88) and any healthcare exposure in the previous 3 months (AOR 1.99, 95% CI 1.14–3.39) (Fig. 2, Supplementary Table 3). For 3GC-R-Kpn colonisation, younger age (AOR 0.59 per 1 year increase, 95% CI 0.47–0.73), lower weight for age Z-score (AOR 0.82 per 1 Z-score increase, 95% CI 0.71–0.95), and inpatient care in the previous 3 months (AOR 2.40, 95% CI 1.32–4.35) increased the odds while current breastfeeding was shown to be protective (AOR 0.51, 95% CI 0.32–0.79) for children under 1 year old (Fig. 2, Supplementary Table 4). For CRE, antibiotic exposure in the previous 4 weeks increased the risk of CRE-Ec colonisation (AOR 9.04, 95% CI 2.56–29.2), while younger age (OR 0.17 per 1 year increase, 95% CI 0.03–0.58) and inpatient exposure (OR 5.90, 95% CI 1.19–24.7) were associated to CRE-Kpn colonisation at enrolment (Fig. 2, Supplementary Tables 5 & 6).

**Fig. 3:**
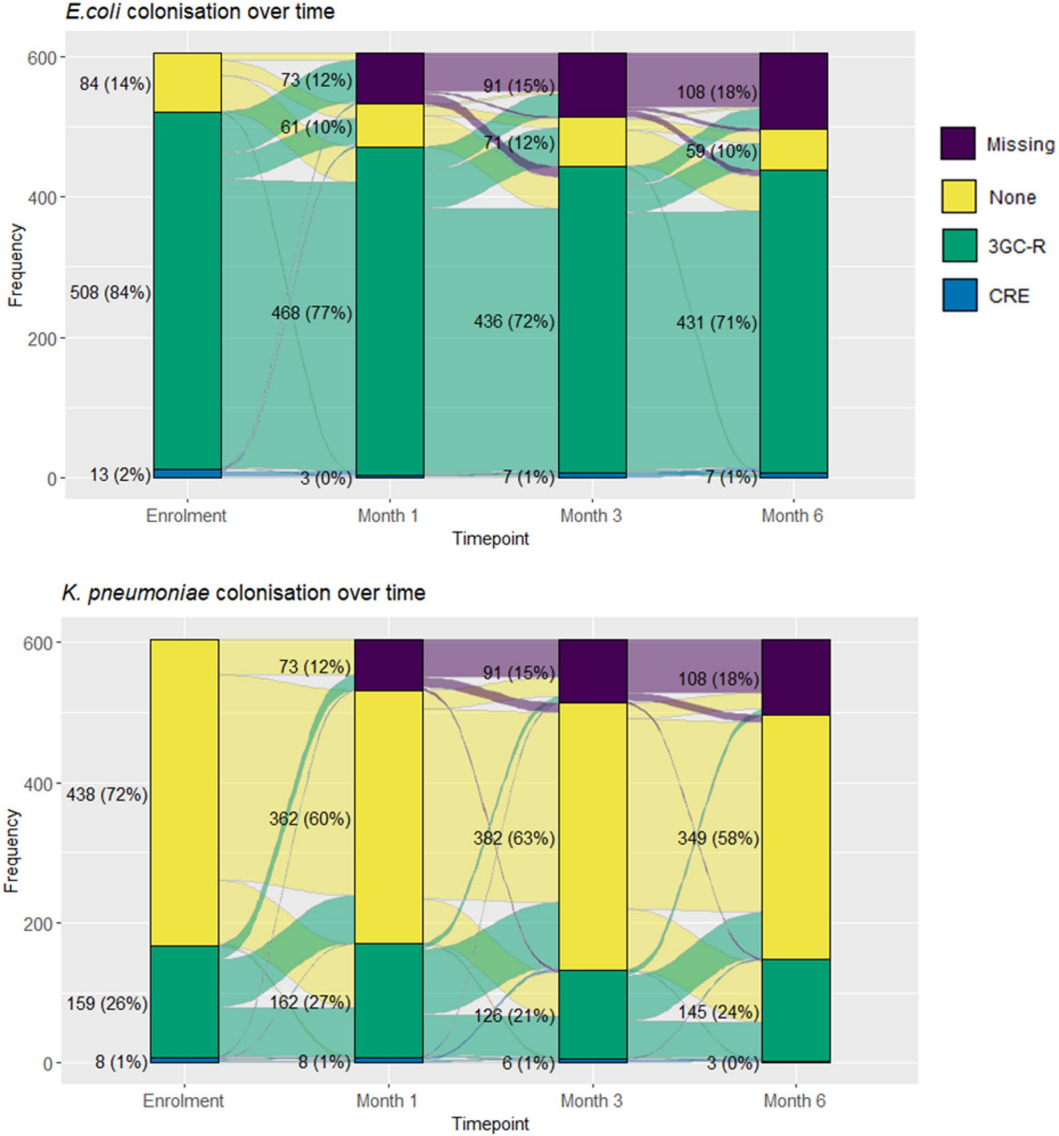
Temporal variation of 3rd -generation cephalosporin resistant (3GC-R) and carbapenem-resistant (CRE) *Escherichia coli* and *Klebsiella pneumoniae* gastrointestinal colonisation in children under 5 years old seen at Angkor Hospital for Children, at enrolment and during 6 months follow-up

### Long-term dynamics of 3GC-R-E and CRE colonisation

**Fig. 4:**
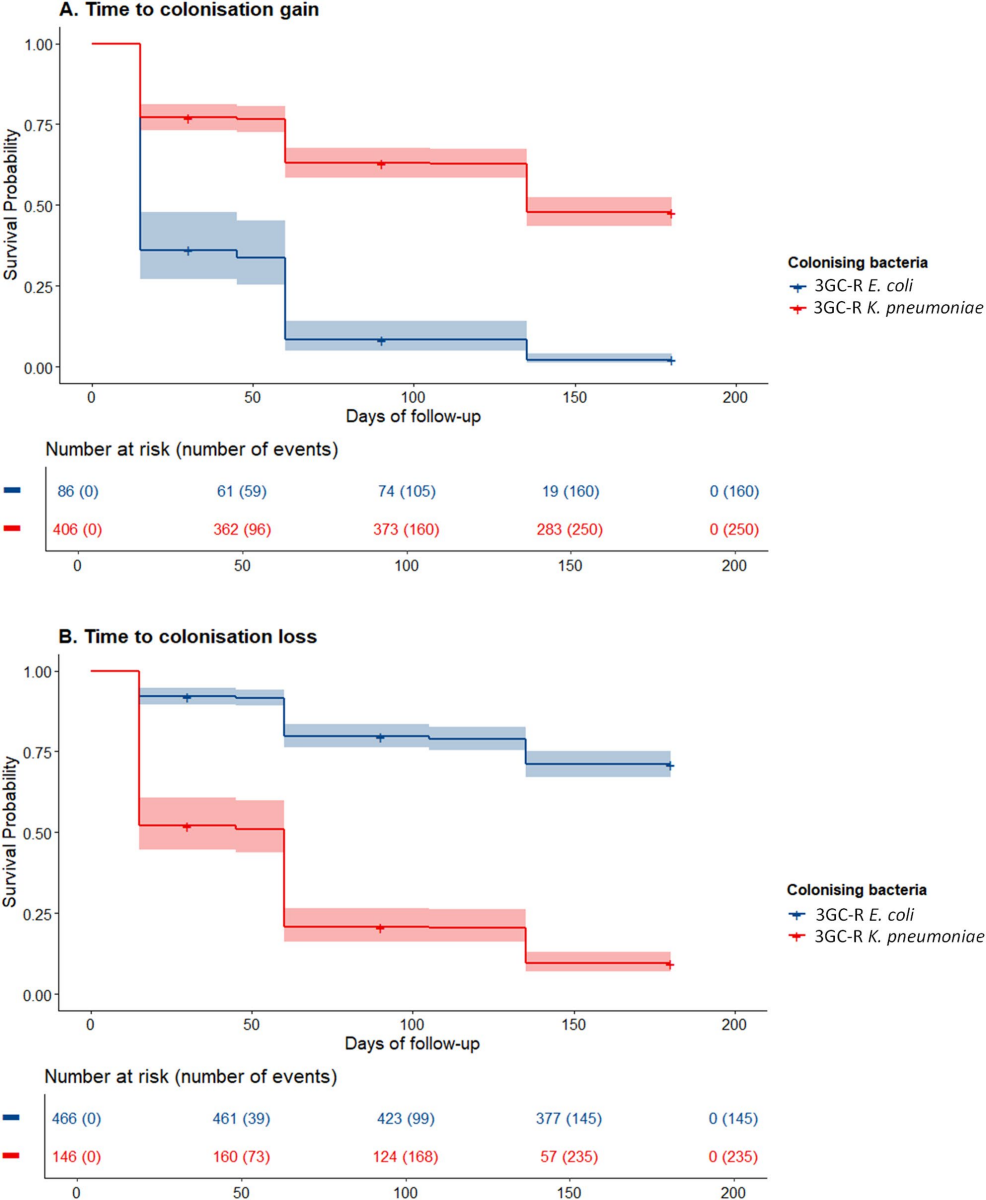
Kaplan Meier curves for (A) time-to-gain and (B) time-to loss for gastrointestinal colonisation of 3rd -generation cephalosporin-resistant (3GC-R) producing *Escherichia coli* and *Klebsiella pneumoniae*, in children under 5 years old seen at Angkor Hospital for Children, at enrolment and during 6 months follow-up

Of the 605 children enrolled in the study, 53 (8.8%) missed all of the follow-up visits with no other relevant baseline differences, and were excluded from the longitudinal analysis (Fig. 1; Table 1). 3GC-R-E colonisation proportions remained quite stable over the 6-month follow-up, ranging between 84 and 88% and for Ec and 25–30% for Kpn (of available samples) (Fig. 3, Supplementary Table 2). However, there were changes in colonisation status for individual children, especially for 3GC-R-Kpn colonisation. For 3GC-R-Ec, at each follow-up visit, 86–92% of those previously colonised remained colonised and 71–80% of those non-colonised became colonised. For 3GC-R-Kpn, at each visit, 38–50% of those previously colonised remained colonised and 19–24% of those non-colonised became colonised. Median time to colonisation gain was 15 days (IQR: 15–15 days) and 135 days (IQR: 135-NA days), respectively, for 3GC-R-Ec and Kpn, while mean time to colonisation loss was undefined for 3GC-R-Ec (Kaplan-Meier curve does not cross 50%) and 60 days (IQR: 15–60 days) for 3GC-R-Kpn (Fig. 4). Overall antibiotic susceptibilities were similar to baseline results (Supplementary Table 2), however there was considerable intra-individual temporal variation (Supplementary Fig. 2).

### Effect of antibiotic and healthcare exposure on 3GC-R-E colonisation dynamics

There were 160 3GC-R-Ec new colonisations (‘gains’) out of 218 potential events and 145 de-colonisations (‘losses’) out of 1325 potential events during the 6 months follow-up (Table 2; Fig. 4). Overall, per visit, 377 children (18%) reported previous antibiotic use, 39 (1.8%) 3rd generation cephalosporin exposure, 1918 (89%) previous healthcare exposure and 287 (13%) inpatient exposure. Neither healthcare nor antibiotic exposure during this time were associated with time-to-gain or time-to-loss for 3GC-R-Ec colonisation (Table 2). For 3GC-R-Kpn, there were 250 colonisation gains out of 1118 potential events and 235 colonisation losses out of 425 potential events during the 6 months follow-up (Table 2; Fig. 4). Reported previous exposures were similar to 3GC-R-Ec (Supplementary Table 7). Inpatient (Hazard Ratio, HR: 1.42, 95% CI: 1.03–1.96), any antibiotic (HR: 1.40, 95% CI: 1.05–1.89), and 3rd generation cephalosporin exposure (HR: 2.06, 95% CI: 1.12–3.79) were associated with a shorter time to colonisation gain, while any healthcare (HR: 0.68, 95% CI: 0.48–0.94), and inpatient exposure (HR: 0.66, 95% CI: 0.43-1.00) increased the time to colonisation loss, after adjusting for potential baseline confounders (Table 2). 3GC-R: 3rd-generation cephalosporin-resistant.

**Table 2:** Cox proportional hazard regression analysis for effect of healthcare and antibiotic exposure on time-to-gain and time-to-loss of 3rd-generation cephalosporin-resistant (3GC-R) *Escherichia coli* and *Klebsiella pneumoniae* gastrointestinal colonisation during 6-month follow-up in children under 5 years old seen at Angkor Hospital for Children

	Time to gain				Time to loss	
	aHR*	95% CI	*p*-value	aHR*	95% CI	*p*-value
3GC-R -* E. coli*	(*N* = 218 records, 160 events)			(*N* = 1325 records, 145 events)		
Any healthcare exposure	0.80	0.54–1.17	0.246	0.93	0.54–1.60	0.788
Inpatient exposure	1.02	0.68–1.53	0.905	0.79	0.45–1.38	0.409
Any antibiotic exposure	0.76	0.52–1.11	0.157	0.91	0.60–1.38	0.653
3rd gen cephalosporin exposure	0.73	0.20–2.70	0.643	0.73	0.20–2.70	0.643
3GC-R -* K. pneumoniae*	(*N* = 1118 records, 250 events)			(*N* = 425 records, 235 events)		
Any healthcare exposure	1.40	0.83–2.38	0.210	0.68	0.48–0.94	0.022
Inpatient exposure	1.42	1.03–1.96	0.031	0.66	0.43-1.00	0.048
Any antibiotic exposure	1.40	1.05–1.89	0.023	0.79	0.59–1.07	0.132
3rd gen cephalosporin exposure	2.06	1.12–3.79	0.019	0.70	0.30–1.59	0.388

* Adjusted for age, sex, toilet inside the house, hand washing basin in toilet, weight-for-age Z score and current breastfeeding.

CI: confidence interval; 3GC-R: 3rd -generation cephalosporin-resistant; aHR: adjusted hazard ratio.

## Discussion

This prospective cohort study has detected high AMR Enterobacterales carriage proportions in the GI tract of Cambodian children attending a healthcare facility. Risk factors for AMR Enterobacterales carriage at presentation to healthcare included male sex (3GC-R-Ec), younger age, and malnourishment (3GC-R and CRE-Kpn), not currently breastfeeding (3GC-R-Kpn), healthcare exposure (3GC-R-Ec and Kpn) and antibiotic exposure (CRE-Ec). 3GC-R-E colonisation proportions remained stable during the 6-month-follow-up, despite changes in colonisation status for individual children. Colonisation dynamics of the two 3GC-R-E differed greatly, with faster colonisation gain and slower loss for 3GC-R-Ec. Persistent healthcare and antibiotic exposure (particularly 3rd generation cephalosporins and hospitalisations) were associated with changes in colonisation dynamics for 3GC-R-Kpn, but not for 3GC-R-Ec.

### Strengths and limitations

This study included a relatively large sample size and collected extensive information on potential factors associated with AMR bacterial carriage. We included 1, 3 and 6-month follow-ups with both colonisation and exposure dynamic data, with a good retention proportion (82%). We used high standard microbiological techniques for bacteria and AMR identification including culture on chromogenic media, MALDI-TOF MS, and automated antimicrobial susceptibility testing. However, this study also presents some limitations. First, information on environmental and previous healthcare and antibiotic exposure were collected through parental questionnaires, which may introduce information bias. Second, we only included 1–59 months-old children attending a single centre in Cambodia for an acute illness. This may limit the generalisability of our findings to other regions, age groups and to children with chronic diseases or very frequent healthcare contact. Third, we only cultured Ec and Kpn using selective media, and therefore could not estimate the number of children colonised by non-3GC-R-E. This should not be an issue for Ec, as colonisation occurs in nearly all children, but it would have been informative to assess the proportion of children colonised by non-3GC-R-Kpn. Fourth, the low colonisation proportions of CRE limited the analysis of associated factors, as reflected by the large confidence intervals. Finally, only one colony per plate was selected so multiple strains with different resistance profiles colonising the same individual [24] would not have been detected. However, a single colony pick from a selective plate is likely to sample the dominant genotype [25] and the detection of a minor carried type would be influenced by the relative proportions [26]. We will anyways perform whole genome sequencing in a future step to study strain-level carriage and to help determine if the isolates identified at two different timepoints in the same child were the same strain or differed, defining gain as the acquisition of a new strain. In fact, we did observe an intra-individual temporal variation in the antibiotic susceptibility patterns of the 3GC-R isolates, indicating that it is possible the same child was colonised by different strains at two consecutive timepoints.

### Findings in relation to other studies

Current 3GC-R-E colonisation rates in our study (88%) were higher than previously reported in this setting (55% of 3GC-R among children < 16 years old in 2012) [4]. However, our study used selective chromogenic agar to identify resistant isolates which is likely more sensitive than the non-selective chromogenic agar and cefpodoxime/imipenem disks previously used [4], and took place in a healthcare setting, so the sampled population may not have been fully representative of the community. In Sub-Saharan Africa, a 2019 meta-analysis reported a much lower pooled ESBL-E colonisation prevalence of 10% (95% CI 1–32%, range 10–60%) in children [27]. Nevertheless, South-East Asia is the region with the highest estimated risk for AMR spread [3]. Most previous studies on ESBL-E colonisation rates in children in Asia have focused on hospitalised patients, especially neonates, hampering the comparison with our estimates from children presenting to healthcare, as carriage rates of ESBL-E increase rapidly after admission [27]. A meta-analysis of children’s infections in Asian studies reported a pooled prevalence of 3GC and carbapenem resistance, respectively, of 73% (95% CI 50–86%) and 15% (95% CI 1–33%) among Ec, and 76% (95% CI 40–92%) and 13% (95% CI 0–46%) among Kpn isolates [28]. Prevalence of CRE colonisation (3%) was low in our setting, though it is a growing problem in neighbouring countries, such as Vietnam (13% CRE colonisation proportion amongst children at admission) [29]. This is aggravated by the lack of access to more extended spectrum and novel antibiotics in LMICs, such as Cambodia [30, 31]. These data emphasise the serious and urgent public health problem of AMR Enterobacterales in Asia.

Most identified risk factors for AMR Enterobacterales colonisation are likely related with gut microbiome health. Reported antibiotic and healthcare exposure, identified as key risk factors for AMR colonisation in children in previous studies [2, 6–8, 27, 32–34], were associated to colonisation with 3GC-R-Ec and Kpn, and CRE-Ec in our analysis. In Cambodia there is a high prevalence of inappropriate antibiotic prescription and use in the community, including broad-spectrum antibiotics [35, 36], and this can result in diminished gut microbiome diversity [37]. Undernutrition appeared to increase the risk of 3GC-R and CRE-Kpn colonisation, even after adjusting for previous healthcare or antibiotic exposure. Undernutrition may affect the gut microbiome composition and is associated with reduced host immunity, therefore increasing the risk of colonisation by pathogenic bacteria [38–40]. Breastfeeding was associated with lower 3GC-R-Kpn colonisation risk in children under 1 year old, as has already been shown in this setting for hospitalised neonates [41]. As with the other risk factors, breastfeeding has also been shown to play a key role in gut microbiome composition and diversity [42–44]. Gut microbiome reduced diversity and altered composition may increase the risk of colonisation with AMR bacteria, which benefit from reduced competition due to the suppression of more robust, antibiotic-sensitive strains [45]. Studying the gut microbiome together with gut AMR bacterial colonisation in children may help decipher potential mechanisms of interaction and help distinguish direct impact of the discussed exposures from broader community-level transmission pressures.

Long term colonisation dynamics of 3GC-R-E in children following healthcare contact have been scarcely studied. Most studies were performed on adults, either during hospitalisation or among returning travellers and with short follow-up times [46–49]. Additionally, some of these studies did not assess 3GC-R-Ec and Kpn colonisation separately [46–49]. Given our findings at baseline, we believe colonisation dynamics of these two Enterobacterales may differ greatly. In previous studies, the mean colonisation duration for ESBL-E was only 30 days for international travellers upon return (ESBL *K .pneumoniae* having the shortest decolonisation time) [46], but was longer for European adults and children who had acquired the colonisation in the community (128 days) [49]. In this study, we showed that 3GC-R-Ec colonisation occurs much faster and remains for longer than Kpn, with shorter time-to-gain time and longer time-to-loss time. This difference is probably due to the much higher baseline colonisation proportion for 3GC-R-Ec compared to Kpn, indicating a high community prevalence of 3GC-R-Ec colonisation. As a consequence, most children already colonised with 3GC-R-Ec at baseline remained colonised during the follow-up and the few that lost colonisation (8–14%), were replaced by children newly colonised, to maintain a similar proportion of colonisation at each timepoint. For 3GC-R-Kpn, however, a larger proportion of those colonised at baseline became decolonised (50–62%), but similar to *E. coli*, the proportion of colonisation at each follow-up visit remained stable. These differences in long term 3GC-R-E colonisation dynamics in children have not been previously described.

The impact of repeated antibiotic and healthcare exposure on the long term 3GC-R-E colonisation dynamics in children is largely unknown. A previous Cambodian study reported that any antibiotic use increased the daily acquisition risk of 3GC-R-Kpn among neonates during hospitalisation [41]. We have now shown that persistent antibiotic exposure and repeated hospitalisations increase the risk of new 3GC-R-Kpn colonisation episodes over time after a first presentation to healthcare, while repeated healthcare exposures increase the time to colonisation loss. A similar study in Malawian adults also showed that repeated hospitalisations increased 3GC-R-E colonisation risk over time, and that persistent antibiotic exposure prolonged colonisation by reducing colonisation loss [48]. However, in the present study, repeated healthcare and antibiotic exposure were not associated with long term 3GC-R-Ec colonisation gain or loss. This may be due to the higher proportion of children in the community in our setting that are colonised with 3GC-R-Ec compared to Kpn [4], which would also explain the higher proportion of new colonisation episodes occurring at each visit over time for 3GC-R-Ec compared to Kpn in our study (71–80% vs. 19–24%).

### Implications for practice and future research

The high rate of AMR bacterial gut colonisation found in our setting confirms the urgent need to reduce this global health problem. Some identified risk factors are modifiable, and point towards interventions that may reduce AMR carriage. Public health policies to promote and protect breastfeeding practices and to reduce undernutrition may contribute to reducing AMR bacterial gut colonisation. Antimicrobial stewardship and reducing healthcare contact, at least with large healthcare centres, are two other key interventions. Digital health algorithms have shown to reduce antibiotic prescription in children in community settings in Tanzania, with no increased clinical failure [50]. Such tools may not only reduce antibiotic use but also unnecessary referrals to larger hospitals, and their extended use should be now assessed in large multicentre clinical trials and in similar LMIC settings in other regions, such as South-East Asia. Adding bacterial colonisation analysis to these studies will enable the study of their impact on AMR colonisation. Interventions modifying gut microbiome diversity and composition, may also reduce AMR gut colonisation in children. The effects of probiotics warrant investigation in robust clinical trials, based on early observational evidence suggesting reduced colonisation with AMR bacteria [8]. Finally, advanced techniques such as targeted metagenomic sequencing from cultured specimens, may increase bacterial colonisation detection [51] and enable longitudinal changes of specific resistomes to be studied [52], an avenue we are currently pursuing.

### Conclusion

Carriage of 3GC-R Enterobacterales is prevalent in Cambodian children. This may limit effective antibiotic choices if followed by invasive infections. In LMICs with high 3GC-R-E colonisation rates, healthcare and antibiotic exposure reduction may prevent 3GC-R-Kpn colonisation with further prevention strategies needed for 3GC-R-Ec colonisation.

## Supplementary Information

Below is the link to the electronic supplementary material.

Supplementary Material 1

Supplementary Material 2

## Data Availability

The datasets used and/or analysed during the current study are available from the Mahidol-Oxford Tropical Medicine Research Unit Data Access Committee on reasonable request (https://www.tropmedres.ac/units/moru-bangkok/bioethics-engagement/data-sharing).

## Abbreviations

3GC-R: Third generation cephalosporin-resistant
3GC-R-Ec: Third generation cephalosporin-resistant *Escherichia coli*
3GC-R-Kpn: Third generation cephalosporin-resistant *Klebsiella pneumoniae*
AHC: Angkor Hospital for Children
AMR: Antimicrobial resistance / resistant
AOR: Adjusted odds ratio
BMI: Body mass index
CRE: Carbapenem resistant Enterobacterales
ESBL(-E): Extended-spectrum beta-lactamase producing (Enterobacterales)
GI: Gastrointestinal
HR: Hazard ratio
IPD: Inpatient
IQR: Interquartile range
LMICs: Low- and middle-income countries
MDR: Multi-drug resistant
OPD: Outpatient
OR: Odds ratio
RS: Rectal swab
SE: Standard error
