# Supplementary material for: Antibiotic and healthcare exposure impact on dynamics of third-generation cephalosporin-resistant Enterobacterales colonisation in Cambodian children: a six-month cohort study
Source: Antimicrob Resist Infect Control. 2026 Apr 22;15:83. doi: 10.1186/s13756-026-01754-3 (PMC13238065; doi:10.1186/s13756-026-01754-3)
Supplement: Supplementary file 2 — Supplementary Material 2 [file 13756_2026_1754_MOESM2_ESM.docx]

**SUPPLEMENTARY MATERIAL:** **Antibiotic and healthcare exposure impact on dynamics of third-generation cephalosporin-resistant Enterobacterales colonisation in Cambodian children: a six-month cohort study**

***Supplementary methods***

**Multivariable logistic regression models for risk factors for 3^rd^-generation cephalosporin-resistant (3GC-R) *E. coli* and *K. pneumoniae* at presentation to healthcare (baseline)**

Due to few cases and sparse data, comorbidities and surgery in the previous 3 months were not included in multivariable models. Comparison groups were composed of non-3GC-R (irrespective of CRE [carbapenem-resistant enterobacteria] colonisation); and non-CRE (irrespective of 3GC-R colonisation). We constructed two multivariable models for each outcome: one ‘simple’ model including exposures associated with 3GC-R-E or CRE colonisation in univariable analysis with a p-value ≤0.1 and one ‘stepwise’ model using automated stepwise backward selection methods. The final model was selected based on Akaike’s information criterion and simplicity. Age and sex were included as a priori risk factors for all models. Only sex and age were included as covariates in the multivariable model for CRE *E. coli* and only univariate analysis was performed for CRE *K. pneumoniae*, given the few cases and sparse data.

**Multivariable Cox proportional hazard regression models for effect of antibiotic and healthcare exposure on time-to-gain and time-to-loss of 3GC-R *E. coli* and *K. pneumoniae* colonisation during 6-month follow-up**

Each child’s follow-up was divided into 3 periods: enrolment to month 1, month 1 to month 3 and month 3 to month 6. We included only the periods with potential 3GC-R-E colonisation gain for the time-to-gain analysis (non-colonised in previous visit and non-missing posterior follow-up visit, N= 218 periods for 3GC-R *E. coli* and N= 1118 periods for 3GC-R *K. pneumoniae*), and with potential colonisation loss in the time-to-loss analysis (colonised in previous visit and non-missing posterior follow-up visit, N= 1325 periods for 3GC-R *E. coli* and N= 425 periods for 3GC-R *K. pneumoniae*). The Cox regression model was clustered by participant unique ID, to account for the inclusion of more than one period per child. Schoenfeld residuals were used to evaluate the proportional hazards assumption of the Cox proportional hazards model. The assumption held true for all included variables.

**Supplementary Tables and Figures Index**

**Supplementary Table 1:** Characteristics of current disease at enrolment of children under 5 years old presenting at the Angkor Hospital for Children.

**Supplementary Table 2:** Gastrointestinal colonising bacteria and their resistance pattern isolated from children at their presentation to Angkor Hospital for Children and consecutive follow-up visits.

**Supplementary Figure 1:** Gastrointestinal bacterial colonisation rates at enrolment among children under 5 years old presenting at the Angkor Hospital for Children for health care, stratified by inpatient or outpatient location and by age group.

**Supplementary Table 3:** Association between sociodemographic, personal history and environmental factors and gastrointestinal carriage of 3GC-R *Escherichia coli.*

**Supplementary Table 4:** Association between sociodemographic, personal history and environmental factors and gastrointestinal carriage of 3GC-R *Klebsiella pneumoniae.*

**Supplementary Table 5:** Association between sociodemographic, personal history and environmental factors and gastrointestinal carriage of carbapenem-resistant *Escherichia coli*

**Supplementary Table 6:** Association between sociodemographic-personal history and environmental factors and gastrointestinal carriage of carbapenem-resistant *Klebsiella pneumoniae.*

**Supplementary Figure 2:** A) *Escherichia coli* and B) *Klebsiella pneumoniae* gastrointestinal colonisation by number of antibiotic resistance in children under 5 years old seen at Angkor Hospital for Children, at enrolment and during 6 months follow-up.

**Supplementary table 7:** Number and proportion of antibiotic and healthcare exposures per colonisation gain or loss episodes for A) *Escherichia coli* and B) *Klebsiella pneumoniae* during the 6 months follow-up for under 5-year-old children seen at Angkor Hospital for Children.

**Supplementary table 8:** Cox proportional hazard regression analysis for effect of potential confounders on time-to-gain and time-to-loss of 3rd-generation cephalosporin-resistant (3GC-R) *Escherichia coli* and *Klebsiella pneumoniae* gastrointestinal colonisation during 6-month follow-up in children under 5 years old seen at Angkor Hospital for Children.

**Supplementary Table 1:** Disease diagnoses and prescribed antibiotics at enrolment for children under 5 years old presenting at the Angkor Hospital for Children.

|  |  | **N (%)** | | |
| --- | --- | --- | --- | --- |
|  | **N** | **All**  **(N=605)** | **Outpatient**  **(N=460)** | **Inpatient**  **(N=145)** |
| **Current disease:** |  |  |  |  |
| ***Diagnoses*** | 605 |  |  |  |
| Upper respiratory infection |  | 258 (43) | 241 (52) | 17 (12) |
| Gastroenteritis |  | 115 (19) | 57 (12) | 58 (40) |
| Bronchiolitis |  | 60 (10) | 19 (4.1) | 41 (28) |
| Reactive airway disease |  | 42 (7) | 27 (5.9) | 15 (10) |
| Pneumonia |  | 37 (6) | 18 (3.9) | 19 (13) |
| Skin problem/rash |  | 35 (7) | 35 (7.6) | 0 (0) |
| Malnutrition |  | 27 (5) | 20 (4.3) | 7 (4.8) |
| Skin/soft tissue infection |  | 16 (2.6) | 14 (3.0) | 2 (1.4) |
| Dengue |  | 12 (2) | 5 (1.1) | 7 (4.8) |
| Urinary tract infection |  | 8 (1.3) | 2 (0.4) | 6 (4.1) |
| Meningitis |  | 6 (0.9) | 0 (0) | 6 (4.1) |
| Allergic reaction |  | 3 (0.5) | 3 (0.7) | 0 (0) |
| Melioidosis |  | 2 (3) | 0 (0) | 2 (1.4) |
| ***Antibiotic prescribed*** |  |  |  |  |
| *Outpatient* | 460 |  |  |  |
| Amoxicillin |  | 23 (4) | 23 (45) | - |
| Cloxacillin |  | 10 (2) | 10 (20) | - |
| Amoxicillin-clavulanate |  | 9 (1) | 9 (18) | - |
| Ciprofloxacin / ofloxacin |  | 7 (1) | 7 (14) | - |
| Azithromycin / Clarithromycin/ Erythromycin |  | 1 (0.2) | 1 (2.0) | - |
| *Empiric for Inpatients* | 145 |  |  |  |
| Ceftriaxone / cefotaxime |  | 17 (3) | - | 17 (12) |
| Meropenem |  | 1 (0.7) | - | 1 (0.7) |

**Supplementary Table 2:** Number and resistance profiles of gastrointestinal colonising bacteria from children at enrolment and consecutive follow-up visits.

|  | **All isolates** | **Enrolment**  **N = 605** | **Month 1**  **N = 531** | **Month 3**  **N = 514** | **Month 6**  **N = 497** |
| --- | --- | --- | --- | --- | --- |
| ***E. coli*** | **1882** | **525*** | **473^#^** | **445^#^** | **439^##^** |
| AMP-GEN | 440 (23%) | 129 (25%) | 129 (27%) | 89 (20%) | 93 (21%) |
| 3GC | 1882 (100%) | 525 (100%) | 473 (100%) | 445 (100%) | 439 (100%) |
| FQ | 795 (42%) | 232 (44%) | 200 (42%) | 179 (40%) | 184 (37%) |
| Carbapenem | 30 (2%) | 13 (2%) | 3 (0.6%) | 7 (2%) | 7 (2%) |
| MDR | 1304 (69%) | 358 (68%) | 326 (69%) | 303 (68%) | 317 (72%) |
| ***K. pneumoniae*** | **627** | **170**** | **172^#^** | **135**** | **150^#^** |
| AMP-GEN | 110 (18%) | 37 (21%) | 30 (17%) | 23 (17%) | 20 (13%) |
| 3GC | 626 (100%) | 169 (97%) | 172 (100%) | 135 (100%) | 150 (100%) |
| FQ | 317 (51%) | 95 (56%) | 95 (55%) | 56 (41%) | 71 (47%) |
| Carbapenem | 24 (4%) | 8 (4%) | 8 (5%) | 5 (4%) | 3 (2%) |
| MDR | 383 (61%) | 106 (61%) | 112 (65%) | 74 (55%) | 91 (61%) |

3GC: 3^rd^-generation cephalosporin; AMP: ampicillin; FQ: fluoroquinolones; GEN: gentamicin; MDR: multidrug resistant (non-susceptibility to ≥1 agent in ≥3 antimicrobial classes tested, excluding intrinsic resistance).

On occasions, some children were colonised with two strains of *E.coli* or *K.pneumoniae* i.e. one 3GC-R and another CRE. Here are the number of children where this occurred: *: 4, **: 3, #: 2, ##: 1

**Supplementary Figure 1:** Gastrointestinal bacterial colonisation proportions rates among children under 5 years old presenting at the Angkor Hospital for Children for health care, by inpatient or outpatient location and by age group (N= 605).


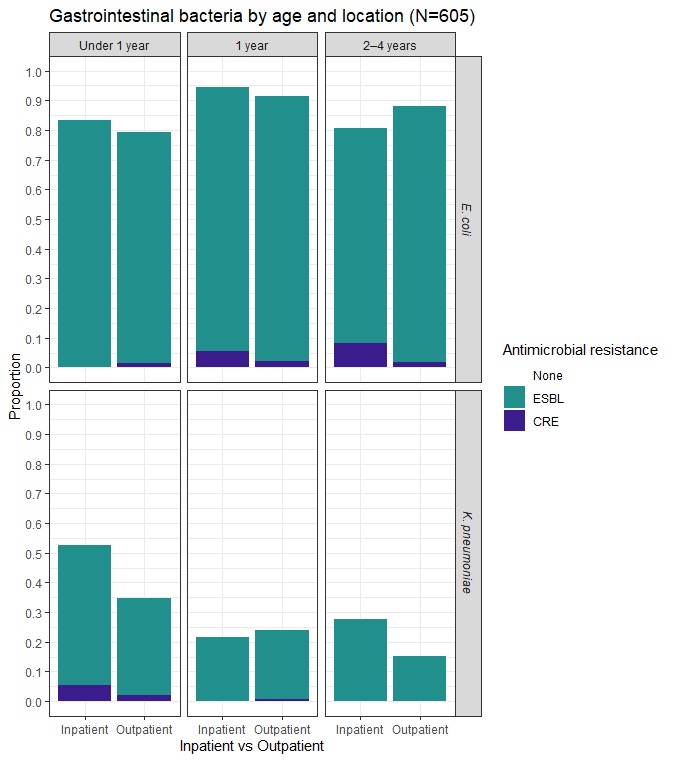


CRE: carbapenem-resistant enterobacteria, 3GC-R: 3^rd^-generation cephalosporin resistant.

**Supplementary Table 3**: Association between sociodemographic, personal history and environmental factors with gastrointestinal carriage of 3GC-R *Escherichia coli* isolated from children at their presentation to Angkor Hospital for Children, Cambodia. (*Adjusted for all the variables in the model).

|  |  | **3GC-R *E. coli*, N (%)** | | **Univariate** | | | **Multivariable** | | |
| --- | --- | --- | --- | --- | --- | --- | --- | --- | --- |
| Characteristic | **N** | **No (N = 93)** | **Yes (N = 512)** | **OR** | **95% CI** | **p-value** | **AOR*** | **95% CI** | **p-value** |
| **Sociodemographics:** |  |  |  |  |  |  |  |  |  |
| Female sex | 605 | 55 (59.1%) | 230 (44.9%) | 0.56 | 0.36-0.88 | 0.012 | **0.56** | **0.36-0.88** | **0.013** |
| Age (years) (median, IQR) | 605 | 1.05 (0.46-2.55) | 1.45 (0.86-2.39) | 1.09 | 0.89-1.33 | 0.419 | 1.09 | 0.89-1.33 | 0.422 |
| **Environment:** |  |  |  |  |  |  |  |  |  |
| People in household (median, IQR) | 605 | 5 (4-7) | 5 (4-7) | 1.02 | 0.93-1.13 | 0.644 |  |  |  |
| Any animal in household | 605 | 65 (69.9%) | 311 (60.7%) | 0.67 | 0.41-1.06 | 0.096 |  |  |  |
| Farm animals | 605 | 36 (38.7%) | 189 (36.9%) | 0.93 | 0.59-1.47 | 0.742 |  |  |  |
| Pets (dogs/cats) | 605 | 51 (54.8%) | 247 (48.2%) | 0.77 | 0.49-1.19 | 0.243 |  |  |  |
| Toilet in the house |  |  |  |  |  |  |  |  |  |
| Inside | 605 | 53 (57.0%) | 281 (54.9%) | 0.92 | 0.59-1.43 | 0.707 |  |  |  |
| With basin for handwashing | 605 | 77 (82.8%) | 417 (81.4%) | 0.91 | 0.49-1.60 | 0.757 |  |  |  |
| School/day care attendance | 605 | 4 (4.3%) | 17 (3.3%) | 0.76 | 0.28-2.70 | 0.636 |  |  |  |
| **Personal history:** |  |  |  |  |  |  |  |  |  |
| BMI Z score (mean, SD) | 538 | -0.53 (1.05) | -0.68 (1.21) | 0.90 | 0.73-1.09 | 0.276 |  |  |  |
| Weight for age Z score (mean, SD) | 605 | -0.79 (1.28) | -0.96 (1.33) | 0.91 | 0.77-1.07 | 0.254 |  |  |  |
| Breastfeeding currently | 605 | 33 (35.5%) | 169 (33.0%) | 0.90 | 0.57-1.44 | 0.641 |  |  |  |
| Health care previous 3 months |  |  |  |  |  |  |  |  |  |
| Any healthcare attendance | 605 | 70 (75.3%) | 441 (86.1%) | 2.04 | 1.18-3.44 | 0.009 | **1.99** | **1.14-3.39** | **0.013** |
| Inpatient | 604 | 10 (10.8%) | 48 (9.4%) | 0.86 | 0.43-1.86 | 0.683 |  |  |  |
| Antibiotics previous 4 weeks | 549 | 6 (6.5%) | 35 (6.8% | 1.06 | 0.47-2.88 | 0.892 | 0.88 | 0.38-2.42 | 0.788 |

AOR: Adjusted odds ratio; BMI: body mass index; CI: confidence interval; ESBL: extended spectrum beta-lactamase producer; HC: health care; IQR: interquartile range; OR: Odds ratio; SD: standard deviation.

**Supplementary Table 4:** Association between sociodemographic, personal history and environmental factors with gastrointestinal carriage of 3GC-R *Klebsiella pneumoniae* isolated from children at their presentation to Angkor Hospital for Children, Cambodia. (*Adjusted for all the variables in the model).

|  |  | **3GC-R *K. pneumoniae*, N (%)** | | **Univariate** | | | **Multivariable** | | |
| --- | --- | --- | --- | --- | --- | --- | --- | --- | --- |
| Characteristic | **N** | **No (N = 443)** | **Yes (N = 162)** | **OR** | **95% CI** | **p-value** | **AOR*** | **95% CI** | **p-value** |
| **Sociodemographics:** |  |  |  |  |  |  |  |  |  |
| Female sex | 605 | 213 (48.1%) | 72 (44.4%) | 0.86 | 0.60-1.24 | 0.428 | 0.95 | 0.65-1.39 | 0.795 |
| Age (years) (median, IQR) | 605 | 1.61 (0.90-2.55) | 0.97 (0.61-1.92) | 0.70 | 0.58-0.83 | <0.001 | **0.59** | **0.47-0.73** | **<0.001** |
| **Environment:** |  |  |  |  |  |  |  |  |  |
| People in household (median, IQR) | 605 | 5 (4-7) | 5 (4-6) | 0.96 | 0.88-1.03 | 0.271 |  |  |  |
| Any animal in household | 605 | 284 (64.1%) | 92 (56.8%) | 0.74 | 0.51-1.06 | 0.101 | 0.76 | 0.52-1.12 | 0.164 |
| Farm animals | 605 | 169 (38.1%) | 56 (34.6%) | 0.86 | 0.59-1.24 | 0.420 |  |  |  |
| Pets (dogs/cats) | 605 | 226 (51.0%) | 72 (44.4%) | 0.77 | 0.53-1.10 | 0.153 |  |  |  |
| Toilet in the house |  |  |  |  |  |  |  |  |  |
| Inside | 605 | 250 (56.4%) | 84 (51.9%) | 0.83 | 0.58-1.19 | 0.316 |  |  |  |
| With basin for handwashing | 605 | 353 (79.7%) | 141 (87.0%) | 1.71 | 1.04-2.92 | 0.040 | 1.66 | 0.98-2.90 | 0.067 |
| School/day care attendance | 605 | 16 (3.6%) | 5 (3.1%) | 0.85 | 0.27-2.21 | 0.755 |  |  |  |
| **Personal history:** |  |  |  |  |  |  |  |  |  |
| BMI Z score (mean, SD) | 538 | -0.62 (1.18) | -0.76 (1.20) | 0.91 | 0.77-1.07 | 0.245 |  |  |  |
| Weight for age Z score (mean, SD) | 605 | -0.87 (1.34) | -1.09 (1.26) | 0.88 | 0.76-1.01 | 0.076 | **0.82** | **0.71-0.95** | **0.011** |
| Breastfeeding currently | 605 | 151 (34.1%) | 51 (31.5%) | 0.89 | 0.60-1.30 | 0.548 | **0.51** | **0.32-0.79** | **0.003** |
| Health care previous 3 months |  |  |  |  |  |  |  |  |  |
| Any healthcare attendance | 605 | 370 (83.5%) | 141 (87.0%) | 1.32 | 0.80-2.28 | 0.292 |  |  |  |
| Inpatient | 604 | 29 (6.6%) | 29 (17.9%) | 3.11 | 1.79-5.40 | <0.001 | **2.40** | **1.32-4.35** | **0.004** |
| Antibiotics previous 4 weeks | 549 | 24 (5.4%) | 17 (10.5%) | 2.05 | 1.05-3.90 | 0.031 | 1.56 | 0.74-3.21 | 0.234 |

AOR: Adjusted odds ratio; BMI: body mass index; CI: confidence interval; ESBL: extended spectrum beta-lactamase producer; HC: health care; IQR: interquartile range; OR: Odds ratio; SD: standard deviation.

**Supplementary Table 5:** Association between sociodemographic, personal history and environmental factors with gastrointestinal carriage of carbapenem-resistant *Escherichia coli* isolated from children at their presentation to Angkor Hospital for Children, Cambodia*.* (*Adjusted for age and sex).

|  | **N** | **CRE *E. coli* N (%)** | | **Univariate** | | | **Multivariable** | | |
| --- | --- | --- | --- | --- | --- | --- | --- | --- | --- |
|  |  | **No (N= 592)** | **Yes (N=13)** | **OR** | **95% CI** | **p-value** | **AOR*** | **95% CI** | **p-value** |
| **Sociodemographics:** |  |  |  |  |  |  |  |  |  |
| Female sex | 605 | 278 (47.0%) | 7 (53.8%) | 1.32 | 0.43-4.14 | 0.624 | 1.26 | 0.41-3.98 | 0.680 |
| Age (years) (median, IQR) | 605 | 1.67 (1.13) | 2.26 (1.48) | 1.49 | 0.95-2.29 | 0.070 | 1.48 | 0.95-2.28 | 0.074 |
| **Environment:** |  |  |  |  |  |  |  |  |  |
| People in household (median, IQR) | 605 | 5 (4-7) | 5 (4-6) | 1.01 | 0.77-1.24 | 0.964 |  |  |  |
| Any animal in household | 605 | 366 (61.8%) | 10 (76.9%) | 2.06 | 0.62-9.25 | 0.277 |  |  |  |
| Farm animals | 605 | 219 (37.0%) | 6 (46.2%) | 1.46 | 0.46-4.45 | 0.501 |  |  |  |
| Pets (dogs/cats) | 605 | 291 (49.2%) | 7 (53.8%) | 1.21 | 0.40-3.79 | 0.738 |  |  |  |
| Toilet |  |  |  |  |  |  |  |  |  |
| Inside house | 598 | 328 (55.4%) | 6 (46.2%) | 0.69 | 0.22-2.10 | 0.509 |  |  |  |
| With basin for handwashing | 598 | 486 (82.1%) | 8 (61.5%) | 0.35 | 0.11-1.17 | 0.070 | 0.35 | 0.11-1.17 | 0.068 |
| School/day care attendance | 605 | 20 (3.4%) | 1 (7.7%) | 2.38 | 0.13-13.0 | 0.415 |  |  |  |
| **Personal history:** |  |  |  |  |  |  |  |  |  |
| BMI Z score (mean, SD) | 538 | -0.65 (1.19) | -0.77 (1.14) | 0.92 | 0.58-1.49 | 0.737 |  |  |  |
| Weight for age Z score (mean, SD) | 605 | -0.92 (1.33) | -1.21 (1.30) | 0.85 | 0.56-1.28 | 0.442 |  |  |  |
| Breastfeeding currently | 605 | 199 (33.6%) | 3 (23.1%) | 0.59 | 0.13-1.96 | 0.430 |  |  |  |
| *Health care previous 3 months* |  |  |  |  |  |  |  |  |  |
| Any HC attendance | 605 | 499 (84.3%) | 12 (92.3%) | 2.24 | 0.43-41.0 | 0.442 |  |  |  |
| In-hospital stay | 604 | 56 (9.5%) | 2 (15.4%) | 1.74 | 0.26-6.68 | 0.480 |  |  |  |
| Antibiotics previous 4 weeks | 549 | 36 (6.1%) | 5 (38.5%) | **9.65** | **2.80-30.5** | **<0.001** | **9.04** | **2.56-29.2** | **<0.001** |

AOR: Adjusted odds ratio; BMI: body mass index; CI: confidence interval; CRE: carbapenem-resistant enterobacteria; HC: health care; IQR: interquartile range; OR: Odds ratio; SD: standard deviation.

**Supplementary table 6:** Association between sociodemographic-personal history and environmental factors with gastrointestinal carriage of carbapenem-resistant *Klebsiella pneumoniae* isolated from children at their presentation to Angkor Hospital for Children, Cambodia*.*

|  |  | **CRE *K. pneumoniae* N (%)** | | **Univariate** | | |
| --- | --- | --- | --- | --- | --- | --- |
|  | **N** | **No (N= 597)** | **Yes (N=8)** | **OR** | **95% CI** | **p-value** |
| **Sociodemographics:** |  |  |  |  |  |  |
| Female sex | 605 | 282 (47.2%) | 3 (37.5%) | 0.67 | 0.14-2.76 | 0.586 |
| Age (years) (median, IQR) | 605 | 1.42 (0.80-2.45) | 0.61 (0.56-0.74) | **0.17** | **0.03-0.58** | **0.020** |
| **Environment:** |  |  |  |  |  |  |
| People in household (median-IQR) | 605 | 5 (4-7) | 6 (4-6) | 1.05 | 0.76-1.34 | 0.725 |
| Any animal in household | 605 | 369 (61.8%) | 7 (87.5%) | 4.33 | 0.76-81.1 | 0.172 |
| Farm animals | 605 | 223 (37.4%) | 2 (25.0%) | 0.56 | 0.08-2.45 | 0.479 |
| Pets (dogs/cats) | 605 | 292 (48.9%) | 6 (75.0%) | 3.13 | 0.72-21.5 | 0.164 |
| Toilet |  |  |  |  |  |  |
| Inside house | 598 | 330 (55.3%) | 4 (50.0%) | 0.81 | 0.19-3.45 | 0.766 |
| With basin for handwashing | 598 | 487 (81.6%) | 7 (87.5%) | 1.58 | 0.28-29.7 | 0.670 |
| School/day care attendance | 605 | 20 (3.4%) | 1 (12.5%) | 4.12 | 0.22-24.8 | 0.195 |
| **Personal history:** |  |  |  |  |  |  |
| BMI Zscore (mean-SD) | 538 | -0.65 (1.19) | -0.86 (0.90) | 0.87 | 0.48-1.62 | 0.650 |
| Weight for age Zscore (mean-SD) | 605 | -0.92 (1.33) | -1.74 (0.89) | 0.62 | 0.39-1.05 | 0.063 |
| Breastfeeding currently | 605 | 198 (33.2%) | 4 (50.0%) | 2.02 | 0.47-8.60 | 0.325 |
| *Health care previous 3 months* |  |  |  |  |  |  |
| Any HC attendance | 605 | 505 (84.6%) | 6 (75.0%) | 0.55 | 0.12-3.77 | 0.464 |
| In-hospital stay | 604 | 55 (9.2%) | 3 (37.5%) | **5.90** | **1.19-24.7** | **0.017** |
| Antibiotics previous 4 weeks  BMI: body mass index; CI: confidence interval; CRE: carbapenemase-resistant enterobacteria; HC: health care; IQR: interquartile range; OR: Odds ratio; SD: standard deviation. | 549 | 40 (6.7%) | 1 (12.5%) | 1.99 | 0.11-11.6 | 0.525 |

**Supplementary Figure 2:** Sankey graph of temporal variation of A) *Escherichia coli* and B) *Klebsiella pneumoniae* gastrointestinal colonisation, by the number of antibiotic agents resistant to, in children under 5 years old seen at Angkor Hospital for Children, at enrolment and during 6 months follow-up.


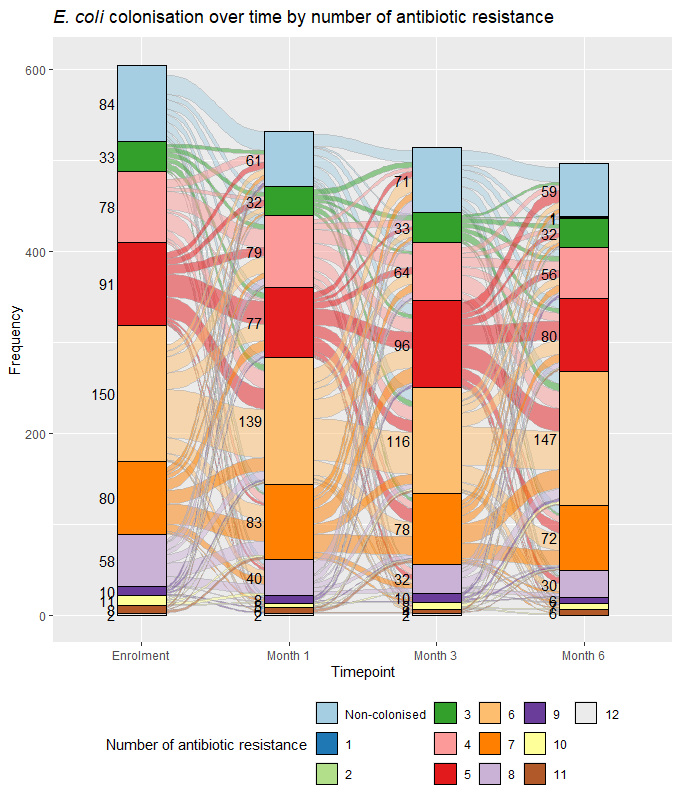


A)

**
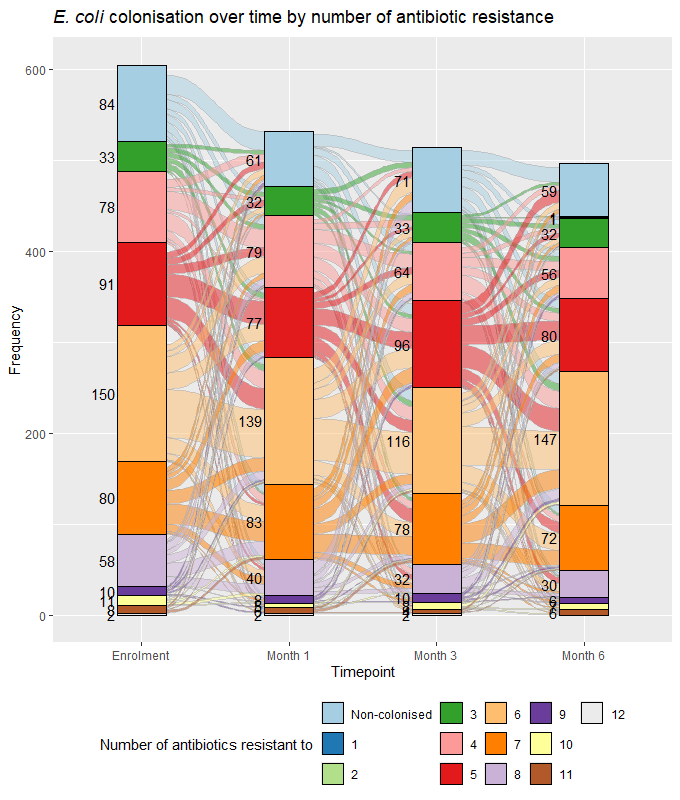
**


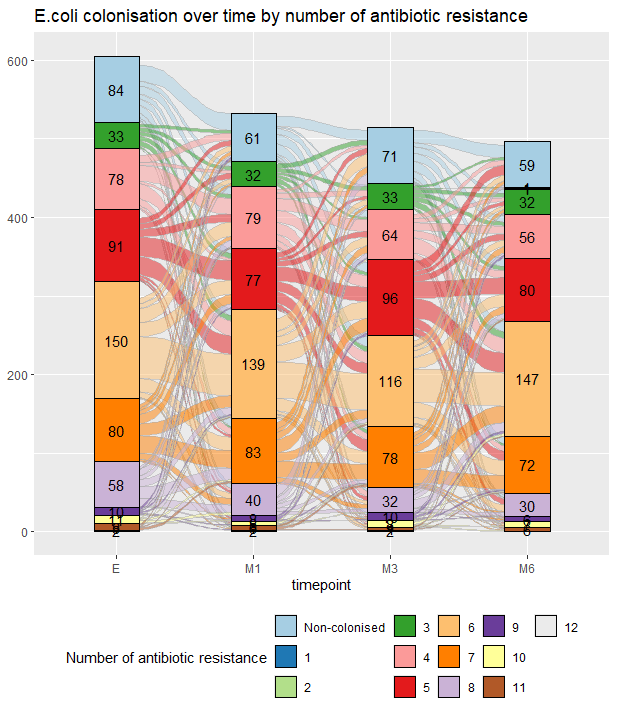


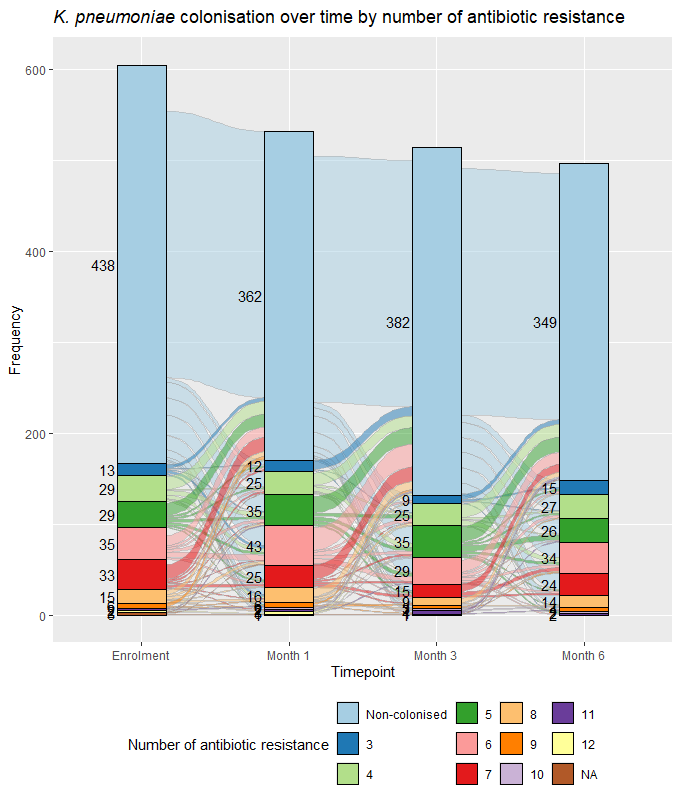


B)

**
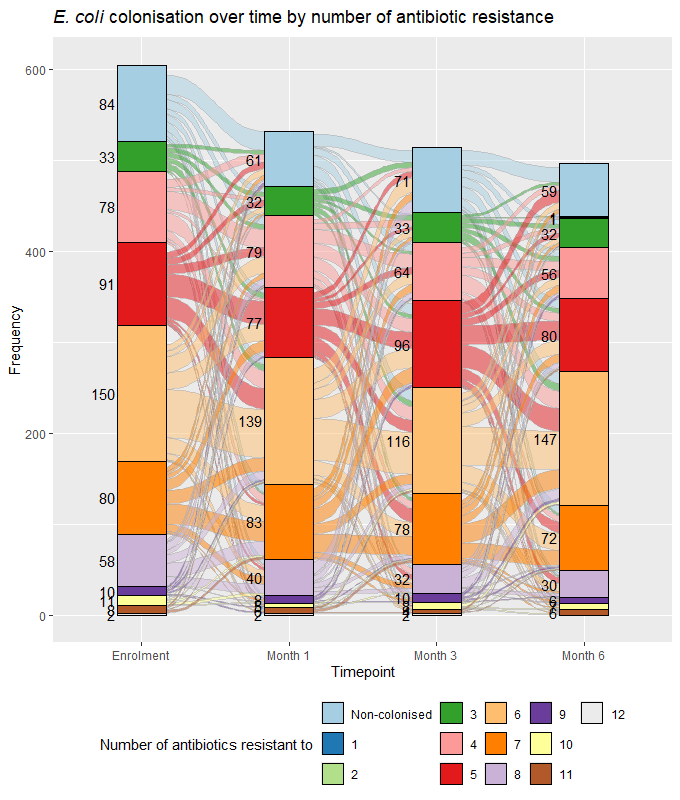
**


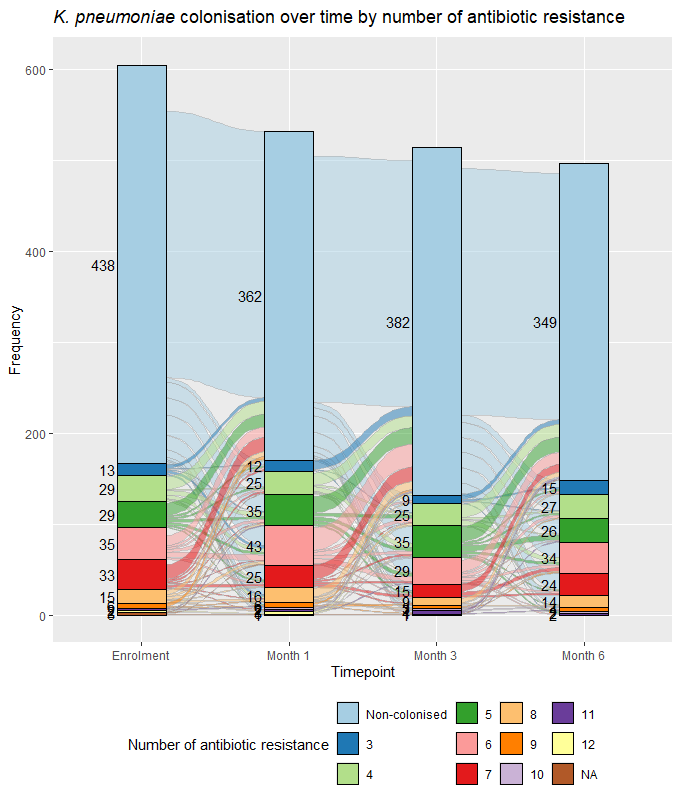


**Supplementary table 7:** Number and proportion of antibiotic and healthcare exposures per colonisation gain or loss episodes for 3GC-R A) *Escherichia coli* and B) *Klebsiella pneumoniae* during the 6 months follow-up for under 5-year-old children seen at Angkor Hospital for Children.

1. **3GC-R *Escherichia coli***

|  | **Colonisation gain episodes** | | | **Colonisation loss episodes** | | |
| --- | --- | --- | --- | --- | --- | --- |
| Exposures | **Overall**  (N = 218) | **No gain**  (N = 58) | **Gain**  (N = 160) | **Overall**  (N = 1325) | **No loss**  (N = 1180) | **Loss**  (N = 145) |
| Any antibiotic | 41 (19%) | 14 (24%) | 27 (17%) | 295 (22%) | 266 (23%) | 29 (20%) |
| Cephalosporin 3rd gen | 5 (2.3%) | 2 (3.4%) | 3 (1.9%) | 30 (2.3%) | 29 (2.5%) | 1 (0.7%) |
| Any healthcare | 200 (92%) | 56 (97%) | 144 (90%) | 1207 (91%) | 1078 (91%) | 129 (89%) |
| Inpatient | 35 (16%) | 10 (17%) | 25 (16%) | 194 (15%) | 179 (15%) | 15 (10%) |

1. **3GC-R *Klebsiella pneumoniae***

|  | **Colonisation gain episodes** | | | **Colonisation loss episodes** | | |
| --- | --- | --- | --- | --- | --- | --- |
| Exposures | **Overall**  (N = 1181) | **No gain**  (N = 868) | **Gain**  (N = 250) | **Overall**  (N = 425) | **No loss**  (N = 190) | **Loss**  (N = 235) |
| Any antibiotic | 232 (21%) | 164 (19%) | 68 (27%) | 104 (24%) | 53 (28%) | 51 (22%) |
| Cephalosporin 3rd gen | 19 (1.7%) | 10 (1.2%) | 9 (3.6%) | 16 (3.8%) | 10 (5.3%) | 6 (2.6%) |
| Any healthcare | 1017 (91%) | 783 (90%) | 234 (94%) | 390 (92%) | 181 (95%) | 209 (89%) |
| Inpatient | 157 (14%) | 108 (12%) | 49 (20%) | 72 (17%) | 42 (22%) | 30 (13%) |

3GC-R: 3^rd^ generation cephalosporin resistant

**Supplementary table 8:** Cox proportional hazard regression analysis for effect of potential confounders on time-to-gain and time-to-loss of 3rd-generation cephalosporin-resistant (3GC-R) *Escherichia coli* and *Klebsiella pneumoniae* gastrointestinal colonisation during 6-month follow-up in children under 5 years old seen at Angkor Hospital for Children.

|  | **Time to gain** | | |  | **Time to loss** |  |
| --- | --- | --- | --- | --- | --- | --- |
|  | **HR** | **95% CI** | **p-value** | **HR** | **95% CI** | **p-value** |
| **3GC-R *- E. coli*** | (N= 218 records, 160 events) | | | (N= 1325 records, 145 events) | | |
| Age (years) | 1.07 | 0.96, 1.20 | 0.241 | 1.30 | 1.13, 1.49 | <0.001 |
| Female sex | 0.90 | 0.66, 1.24 | 0.525 | 1.36 | 0.98, 1.88 | 0.066 |
| Breastfeeding currently | 0.62 | 0.43, 0.88 | 0.008 | 0.40 | 0.26, 0.61 | <0.001 |
| Toilet inside house | 0.71 | 0.51, 0.97 | 0.030 | 1.23 | 0.88, 1.71 | 0.229 |
| Handwashing basin in toilet | 1.19 | 0.79, 1.78 | 0.401 | 1.26 | 0.81, 1.97 | 0.311 |
| Weight-for-age Z score | 0.97 | 0.86, 1.11 | 0.699 | 0.98 | 0.86, 1.10 | 0.688 |
| **3GC-R *- K. pneumoniae*** | (N= 1118 records, 250 events) | | | (N= 425 records, 235 events) | | |
| Age (years) | 0.87 | 0.78, 0.98 | 0.019 | 1.32 | 1.17, 1.48 | <0.001 |
| Female sex | 1.05 | 0.82, 1.34 | 0.715 | 1.07 | 0.83, 1.38 | 0.624 |
| Breastfeeding currently | 0.79 | 0.61, 1.04 | 0.088 | 1.34 | 1.02, 1.76 | 0.035 |
| Toilet inside house | 1.04 | 0.81, 1.34 | 0.748 | 0.81 | 0.63, 1.05 | 0.116 |
| Handwashing basin in toilet | 0.80 | 0.59, 1.07 | 0.138 | 1.03 | 0.74, 1.43 | 0.868 |
| Weight-for-age Z score | 0.92 | 0.84, 1.02 | 0.118 | 0.95 | 0.86, 1.04 | 0.268 |

3GC-R: 3^rd^ generation cephalosporin resistant; CI: confidence interval; HR: hazard ratio.
