# Supplementary material for: Antibiotic and healthcare exposure impact on dynamics of third-generation cephalosporin-resistant Enterobacterales colonisation in Cambodian children: a six-month cohort study
Source: Antimicrob Resist Infect Control. 2026 Apr 22;15:83. doi: 10.1186/s13756-026-01754-3 (PMC13238065; doi:10.1186/s13756-026-01754-3)
Supplement: Supplementary file 1 — Supplementary Material 1 [file 13756_2026_1754_MOESM1_ESM.docx]

To the editor:

Thank you for giving us the opportunity to submit a revised version of this manuscript. We give a point-by-point response to the reviewers’ comments below. We show the comments in plain text and our responses in blue, with newly added text to the manuscript *in italics and underlined*. We indicate the specific location of changes in the clean, revised version of the text using page and line numbers. In addition to the clean, revised version of the manuscript, we submit a version with tracked changes, so that all changes from the original text and display items are easily noted.

Reviewer 1
For the first part of results section, it is important to mention the underlying condition/diagnosis for which the antibiotics were given at first place before the current study was conducted. Also, what were the culture/ susceptibility results then?

**Reply:** We agree with the reviewer that it would have been informative to have this information available, but it was not part of the baseline questionnaire administered to the participant’s parent/legal representative. Antibiotics may have been prescribed by another heath centre, community healer or pharmacy the diagnosis at that visit, even if collected, may not have been accurate. Also, it would have been difficult for the child carers to remember the exact diagnosis. Similarly, we do not have any culture results from previous visits that did not take place at Angkor Hospital for Children. Most health centres and even private clinics do not perform culture and susceptibility testing.

We have explained this briefly in the methods section, page 8, lines 181-184:

*“Potential risk factors for colonisation with AMR bacteria were selected based on previous literature. These included data from the questionnaires: sociodemographic information (age, sex), environmental exposures (pets and farm animals, number of people in the household, access to a toilet inside the house and handwashing basin within 2 meters of the toilet, school or day care attendance), current breastfeeding, comorbidities, healthcare exposure in the previous 3 months (any healthcare consultation and hospital admission), and antibiotic use in the previous 4 weeks (shorter period to avoid recall bias). No specific information on diagnosis from previous healthcare contacts were collected, as these may have taken place outside of AHC and parent/legal representative recall may have been unreliable.”*

What is the possible explanation for the variation in gain of colonization time in *E. coli* vs *K. pneumoniae*?

**Reply:** This is an important finding to highlight from our study. As we have shown, baseline colonisation proportion was much higher for 3GC-R *E. coli* (84%) than for *K. pneumoniae* (26%), which may reflect the high community colonisation prevalence for 3GC-R-*E. coli*. When 3GC-R-Ec presence in the community is so extended and common, it is much easier to become colonised again (shorter time to colonisation gain).

We have added this reflection to the discussion, page 20 lines 519-521:

*“In this study, we showed that 3GC-R-Ec colonisation occurs much faster and remains for longer than Kpn, with shorter time-to-gain time and longer time-to-loss time. This difference is probably due to the much higher baseline colonisation proportion for 3GC-R-Ec compared to Kpn, indicating a high community prevalence of 3GC-R-Ec colonisation.”*

Reviewer 2
Thank you for the opportunity to review your manuscript. The abstract is clear and engaging. The introduction is well framed, though the second paragraph would benefit from clearer organisation. The methods are generally clear – I just wanted more information on the study setting. Results are detailed and informative. The Discussion is insightful with scope to streamline some numerical repetition. Specific comments below:
1.      Title – recommend adding the study design, clarifying that “long-term” refers to the 6-month follow-up to improve indexing accuracy and reduce ambiguity.

**Reply:** We agree with the reviewer and we have modified the title to: *“Antibiotic and healthcare exposure impact on dynamics of third-generation cephalosporin-resistant Enterobacterales colonisation in Cambodian children: a six-month cohort study”*

2.      Abstract – enjoyable to read, with clear rationale, methods, and results. Recommend strengthening the public health implications by explicitly stating how the findings inform AMR control in similar LMIC paediatric settings, and clarifying the relevance for antibiotic stewardship (e.g., implications for empirical prescribing or post-discharge management).

**Reply:** We thank the reviewer for their comment. We have added implications for clinical care and public health strategies in the conclusion as suggested:

*“3GC-R-E carriage is prevalent in Cambodian children, and should be considered for empirical antibiotic treatment of acute infections. Healthcare and antibiotic exposure reduction may prevent 3GC-R-Kpn colonisation with further prevention strategies needed for 3GC-R-Ec colonisation.”*

3.      Introduction – line 104 - recommend clarifying if the Cambodian data reflect hospital-based or community-based populations, as this affects comparability.

**Reply:** The data given are for children attending the same hospital as those in this current study, both at presentation to the hospital and at discharge. We have clarified this:

*“In Cambodia, high colonisation prevalence of extended-spectrum beta-lactamase producing Enterobacterales (ESBL-E) have been documented in children: 55% Escherichia coli / Klebsiella pneumoniae combined in older children presentating to healthcare (4); 92% E. coli and 36% K. pneumoniae in children post-discharge (5).”*

4.      Introduction lines 108 – 121 – this paragraph is informative seemed to lack a clear organising framework (or could just be me). E.g., the adult travel literature may have relevance to Cambodian children – but it isn’t linked clearly and recommend briefly acknowledging generalisability limitations. The transition from risk factors to persistence literature feels abrupt; recommend a clearer signposting sentence to separate acquisition vs persistence evidence.

**Reply:** Thank you for this comment. We have modified the text to make the transition from acquisition to dynamics less abrupt and made additional minor changes to improve the paragraph’s readability:

*“Hospitalisation and broad-spectrum antibiotic use are the main determinants for acquisition of AMR E. coli and K. pneumoniae (3-5). Travel to high prevalence AMR areas, chronic diseases, previous surgeries, invasive procedures, dietary habits and intrafamilial transmission have also been identified as risk factors for AMR bacterial colonisation (3, 6, 7). However, risk factors may vary by region, especially in LMICs. In Cambodian children, hospital admission and intestinal parasites were identified as independent risk factors for AMR E. coli and K. pneumoniae colonisation (1). On the other hand, little is known about the long-term temporal dynamics of carriage after exposure, especially in children. Most data comes from studies on adults returning to Europe after international travel, which have shown a high proportion of gut colonisation with multidrug-resistant Enterobacterales (24-51%), especially when returning from India (72% in a meta-analysis) (8-12). Persistence of colonisation occurred in 36% of the colonised travellers 1 month later (9), and in 5-28% 6 months later (8, 11, 12), associated with a vegetarian diet, travel to Asia, high relative abundance of AMR Enterobacterales, gut microbiota composition and cat ownership (8, 9, 11, 12). However, it is unclear whether we may generalise these findings to Cambodian children. Although genotypically-verified data are relatively sparse, GI duration of colonisation by AMR Enterobacterales in children may be prolonged (17-20).”*

5.      Methods – line 132 indicates the subsection will be about study design and setting, however it largely reads as an ethics statement, with minimal description of the actual setting. Recommend moving ethics approvals to a dedicated Ethics subsection, expanding the setting information to include relevant context about Cambodia (e.g., World Bank income classification, population of children <5), and adding brief detail on Siem Reap and whether its demographics are broadly representative. Also suggest describing key characteristics of Angkor Hospital for Children (catchment, patient volume, referral patterns) to support assessment of generalisability. The STROBE and MICRO reporting guideline statements would be better placed at the end of the Methods.

**Reply:** We thank the reviewer for this comment. We have now provided context about Cambodia, Siem Reap and Angkor Hospital for Children, as suggested, in the first paragraph on the Methods section, page 6 lines 134-140:

*“COMRU-META was a prospective clinical cohort study of children seen at Angkor Hospital for Children (AHC), a non-governmental paediatric referral hospital based in Siem Reap, Cambodia. Cambodia is a lower-middle-income country (16), with an approximate under-five population of 1.8 million children in 2024 (17). Siem Reap province in northwestern Cambodia has a population of around 1 million people (2019), predominantly rural despite its capital being a major cultural and tourism hub, and with age structure similar to national patterns of a young demographic (18). AHC has 73 inpatient beds and provides free primary-to-tertiary care to children under 16 years, with ~100,000 outpatient visits and 5500 admissions annually, irrespective of geographic location.”*

We have left the STROBE and MICRO statements here, so not to add yet another paragraph to the methods section.

6.      Methods – line 144- 145  - the inclusion/exclusion criteria are clear, but rationale for excluding those on systemic antibiotics for the current illness could be briefly stated (risk of culture suppression, etc.).

**Reply:** As the reviewer has stated, the reason for not including children that were already taking systemic antibiotics was to avoid culture suppression and bacterial selection of nasopharyngeal and gastrointestinal colonising bacteria. We wanted to assess the impact of current healthcare contact and antibiotic consumption on the children’s colonisation status, and this would not have been possible if they had already taken antibiotics before the rectal and nasopharyngeal swabs were taken.

We have included this in the methods section, page 7, lines 152-153:

*“Exclusion criteria were: residing outside Siem Reap district, systemic antibiotic use for current illness (to avoid culture suppression and selection impact on colonising bacteria), chronic medical condition (immunosuppression, active chronic infections, active cardiorespiratory conditions, or upper respiratory tract or intestinal tract abnormalities) or AHC prescribed antibiotic started more than 1 hour before enrolment.”*

7.      Methods – line 143 – presumably “non-elective healthcare” includes emergency presentations (this affects representativeness).

**Reply:** As the reviewer points out, we only included children who attended our outpatient or inpatient department for non-elective healthcare, to include only those acutely ill. We excluded children who came for follow-up consultations or elective surgery. We have included a comment in the limitations of the discussion section regarding this.

Discussion section, page 18 lines 453-455:

*“However, this study also presents some limitations. First, information on environmental and previous healthcare and antibiotic exposure were collected through parental questionnaires, which may introduce information bias. Second, we only included 1-59 months-old children attending a single centre in Cambodia for an acute illness. This may limit the generalisability of our findings to other regions, age groups and to children with chronic diseases or very frequent healthcare contact.”*

8.      Methods – good to clarifying the timeframe mismatch (healthcare exposure previous 3 months; antibiotics previous 4 weeks) to justify analytic choices.

**Reply:** We appreciate the importance of this difference. As we have seen in this and previous studies, it is more difficult for carers to remember about specific drugs prescribed than whether they had any healthcare contact or not. We wanted to extend the healthcare exposure to the previous 3 months, as we know that GI duration of colonisation by AMR Enterobacterales in children may be prolonged (as described in the introduction). However, extending the reporting of antibiotic use to the last 3 months would likely have increased recall bias.

We have added a short clarification in the methods section, page 8, lines 181-182:

*“Potential risk factors for colonisation with AMR bacteria were selected based on previous literature. These included data from the questionnaires: sociodemographic information (age, sex), environmental exposures (pets and farm animals, number of people in the household, access to a toilet inside the house and handwashing basin within 2 meters of the toilet, school or day care attendance), current breastfeeding, comorbidities, healthcare exposure in the previous 3 months (any healthcare consultation and hospital admission), and antibiotic use in the previous 4 weeks (shorter period to avoid recall bias).”*

9.      Methods – the analytical approach is appropriate and well-justified. At line 193 for e.g., can the authors specify the criteria for inclusion of risk factors in multivariable models (p-value threshold, clinical relevance).

**Reply:** The initial selection of the studied factors was based in previous literature (first line of “Definitions of risk factors and outcomes” paragraph of the methods section). The development of the multivariable models is described in detail in the supplementary materials (Supplementary methods, page 1). We decided to do this given the length of the current manuscript. If the Editor believes it is necessary to move this detailed description to the main text, we will be happy to do so.

10.     Methods – for missing data, how were missing data were assessed (random vs systematic) and was any imputation attempted?

**Reply:** Missing data was assessed as missing at random, that is why children with missing values were excluded from the analyses. No imputation was attempted. We have included this detail in the methods section, p. 9, lines 207-208:

*“Children with missing values for included variables were excluded from the analyses. No imputation for missing values was attempted.”*

11.     Results – the high enrolment colonisation prevalence is appropriately highlighted; however, recommend explicitly acknowledging the potential effect of the hospital-based sampling frame on these proportions

**Reply:** We agree with the fact that this is not a community-based study, as described in the methods. In fact, the first sentence in the discussion that summarises the results of the current study, state that the colonisation proportions are from Cambodian children attending a healthcare facility, page 17, lines 435-436 *(“This prospective cohort study has detected high AMR Enterobacterales carriage proportions in the GI tract of Cambodian children attending a healthcare facility.” ).*

However, rectal swabs were collected at presentation to healthcare, and not during hospitalisation. So the baseline colonisation status was not due to the current healthcare contact. Also, previous studies in this same location already demonstrated a high colonisation prevalence in children and families without recent healthcare exposure (reference 5 of the manuscript).

We have added a comment in the discussion addressing that this study took place in a healthcare setting, and despite taking swabs at presentation, the sampled population may not have been fully representative of the community (p. 18 lines 472-476).:

*“Current 3GC-R-E colonisation rates in our study (88%) were higher than previously reported in this setting (55% of 3GC-R among children <16 years old in 2012) (1). However, our study used selective chromogenic agar to identify resistant isolates which is likely more sensitive than the non-selective chromogenic agar and cefpodoxime/imipenem disks previously used (4), and took place in a healthcare setting, so the sampled population may not have been fully representative of the community.”*

12.     Results- the finding that breastfeeding is protective for K. pneumoniae in children <1 year is important; recommend drawing more attention to it as a potentially modifiable factor.

**Reply:** We thank the reviewer for this comment. We have added a sentence in the discussion section to draw more attention on breastfeeding as a modifiable factor (p. 21, lines 542-544):

*“The high rate of AMR bacterial gut colonisation found in our setting confirms the urgent need to reduce this global health problem. Some identified risk factors are modifiable, and point towards interventions that may reduce AMR carriage. Public health policies to promote and protect breastfeeding practices and to reduce undernutrition may contribute to reducing AMR bacterial gut colonisation. Antimicrobial stewardship and reducing healthcare contact, at least with large healthcare centres, are two other key interventions.”*

13.     Results – for CRE models, wide confidence intervals reflect sparse data; recommend briefly acknowledging this limitation.

**Reply:** We agree, the low colonisation proportion of CRE led to very low numbers, limiting the analysis of risk factors because of sparse data. We have now acknowledged this in the limitations section of the discussion (p. 18, lines 459-460).

*“However, this study also presents some limitations. First, information on environmental and previous healthcare and antibiotic exposure were collected through parental questionnaires, which may introduce information bias. Second, we only included 1-59 months-old children attending a single centre in Cambodia for an acute illness. This may limit the generalisability of our findings to other regions, age groups and to children with chronic diseases or very frequent healthcare contact. Third, we only cultured E. coli and K. pneumoniae using selective media, and therefore could not estimate the number of children colonised by non-3GC-R-E. This should not be an issue for E. coli, as colonisation occurs in nearly all children, but it would have been informative to assess the proportion of children colonised by non-3GC-R-Kpn. Fourth, the low colonisation proportions of CRE limited the analysis of associated factors, as reflected by the large confidence intervals.”*

14.     Results - Loss-to-follow-up of 18% is reasonable but recommend commenting on whether characteristics of those lost differed from completers.

**Reply:** Thank you for the comment. We have realised the description of the follow-up study population was not clear enough. Of the 605 children enrolled at the start of the study, 53 (8.8%) missed **all** the follow-up visits (at month 1, 3 and 6). This cohort we have now defined as ‘lost-to-follow-up’ (LTFU) and they were excluded from the longitudinal analysis. The rest of the children attended at least one of the visits and were therefore included in the longitudinal analysis (time-to-gain and time-to-loss). Some may have missed one visit but came to the next or attended at least the first visit, so also contributed data.

We have modified the Figure 1 and the text in the methods section, p. 14, lines 344-345:

*“Of the 605 children enrolled in the study, 53 (8.8%) missed all of the follow-up visits* *with no other relevant baseline differences, and were excluded from the longitudinal analysis (Figure 1 and Table 1).”*

We have also modified Table 1 to stratify the baseline characteristics by LTFU vs non-LTFU, showing there were no relevant differences.

15.     Discussion – the contrast between E. coli and K. pneumoniae colonisation dynamics is a major contribution and should be emphasised more strongly and earlier.

**Reply:** Thank you for this comment. We agree that this is an important finding, and we have now included it in the first paragraph of the discussion, within the summary of the main findings (p. 17, lines 441-442):

*“This prospective cohort study has detected high AMR Enterobacterales carriage proportions in the GI tract of Cambodian children attending a healthcare facility. Risk factors for AMR Enterobacterales carriage at presentation to healthcare included male sex (3GC-R-Ec), younger age, and malnourishment (3GC-R and CRE K. pneumoniae), not currently breastfeeding (3GC-R-Kpn), healthcare exposure (3GC-R-Ec and K. pneumoniae) and antibiotic exposure (CRE E. coli). 3GC-R-E colonisation proportions remained stable during the 6-month-follow-up, despite changes in colonisation status for individual children. Colonisation dynamics of the two 3GC-R-E differed greatly, with faster colonisation gain and slower loss for 3GC-R-Ec. Persistent healthcare and antibiotic exposure (particularly 3rd generation cephalosporins and hospitalisations) were associated with changes in colonisation dynamics for 3GC-R-Kpn, but not for 3GC-R-Ec.”*

16.     Discussion - Some numerical results are repeated unnecessarily; recommend removing detailed figures already reported in Results.

**Reply:** Thank you for your comment. We have deleted some figures from the discussion that were already reported in the results (for example p.21 lines 515-516). However, we have left some (very few) percentages, to make it easier for the reader when comparing with previous literature.

17.     Discussion- the limitation regarding selective media and single-colony picks is important; recommend commenting briefly on how this may bias estimates (e.g., under detecting strain diversity).

**Reply:** We have acknowledged that the single-colony pick hampered the detection of multiple strains with different resistance profiles. Even though individuals can be colonised by multiple strains, statistically, a single colony pick from a selective plate is likely to sample the dominant genotype. We know from studies on other colonising bacteria such as *Streptococcus pneumoniae*, that detection of a minor carried type would be influenced by the relative proportions. All in all, we plan to explore strain-level carriage using plate-scrape metagenomics and a previously validated bioinformatics pipeline.

We have added this to the discussion, p. 18, lines 461-467:

*“Finally, only one colony per plate was selected so multiple strains with different resistance profiles colonising the same individual would not have been detected. However, a single colony pick from a selective plate is likely to sample the dominant genotype and the detection of a minor carried type would be influenced by the relative proportions. We will anyways perform whole genome sequencing to study strain-level carriage and which will help determine if the isolates identified at two different timepoints in the same child were the same strain or differed, defining gain as the acquisition of a new strain. In fact, we did observe an intra-individual temporal variation in the antibiotic susceptibility patterns of the 3GC-R isolates, indicating that it is possible the same child was colonised by different strains at two consecutive timepoints.”*

18.     Discussion – consider clarifying whether loss to follow-up differed by colonisation or exposure status.

**Reply:** Please refer to our reply to comment number 14.

19.     Discussion - comparisons with older Cambodian data (2012) should clarify whether methodological differences may partly explain higher prevalence.

**Reply:** The 2012 study was performed in the same setting, but as the reviewer stated, using a different methodology. We have acknowledged this in the discussion (p.18, lines 45472-476):

*“Current 3GC-R-E colonisation rates in our study (88%) were higher than previously reported in this setting (55% of 3GC-R among children <16 years old in 2012) (4). However, our study used selective chromogenic agar to identify resistant isolates which is likely more sensitive than the non-selective chromogenic agar and cefpodoxime/imipenem disks previously used (4), and took place in a healthcare setting, so the sampled population may not have been fully representative of the community.”*

20.     Discussion – the argument that undernutrition and breastfeeding relate to microbiome integrity is convincing; however, recommend avoiding over-interpretation given observational design.

**Reply:** We appreciate the suggestion and agree with it. However, we believe the text we had included only stated the results from our study and previous studies, without making further assumptions on role of the gut microbiome. We have added now one more sentence at the end of the paragraph, to emphasise the need for further studies combining gut microbiome analysis with AMR bacteria gut colonisation (discussion, p.19 lines 503-505):

*“Breastfeeding was associated with lower 3GC-R-Kpn colonisation risk in children under 1 year old, as has already been shown in this setting for hospitalised neonates (40). As with the other risk factors, breastfeeding has also been shown to play a key role in gut microbiome composition and diversity (41-43). Gut microbiome reduced diversity and altered composition may increase the risk of colonisation with AMR bacteria, which benefit from reduced competition due to the suppression of more robust, antibiotic-sensitive strains (44). Studying the gut microbiome together with gut AMR bacterial colonisation in children may help decipher potential mechanisms of interaction and help distinguish direct impact of the discussed exposures from broader community-level transmission pressures.”*

21.     Discussion  - digital decision-support tools are relevant, but the connection to Cambodian context may need brief justification.

**Reply:** Thank you for this comment. Rural Cambodia may share many similarities with rural Tanzania, as they are both LMIC with limited access to health-care in rural areas. However, we have modified the sentence to include the need of assessing the use of such digital decision-support tools in other settings, such as Cambodia or South-East Asia. Discussion section, p. 21, lines 548-549:

*“Antimicrobial stewardship and reducing healthcare contact, at least with large healthcare centres, are two other key interventions. Digital health algorithms have shown to reduce antibiotic prescription in children in community settings in Tanzania, with no increased clinical failure (21). Such tools may not only reduce antibiotic use but also unnecessary referrals to larger hospitals, and their extended use should be now assessed in large multicentre clinical trials and in similar LMIC settings in other regions, such as South-East Asia. Adding bacterial colonisation analysis to these studies will enable the study of their impact on AMR colonisation.”*

22.     Discussion – the section on probiotics may overstate current evidence, recommending more cautious framing given limited trial data.

**Reply:** We have modified this section as suggested (p. 21-22, lines 551553):

*“Interventions modifying gut microbiome diversity and composition, may also reduce AMR gut colonisation in children. The effects of probiotics warrant investigation in robust clinical trials, based on early observational evidence suggesting reduced colonisation with AMR bacteria.”*

23.     Discussion - consider noting the need for microbiome-focused studies to disentangle exposure effects from underlying community transmission pressures.

**Reply:** Please refer to the answer to comment number 20

24.     Conclusion - consider highlighting the broader relevance for LMIC paediatric settings beyond Cambodia.

**Reply:** Thank you for the suggestion. We have modified the conclusion accordingly:

*“Carriage of 3GC-R Enterobacterales is prevalent in Cambodian children. This may limit effective antibiotic choices if followed by invasive infections. In LMICs with high 3GC-R-E colonisation rates, healthcare and antibiotic exposure reduction may prevent 3GC-R-Kpn colonisation with further prevention strategies needed for 3GC-R-Ec colonisation.”*

Reviewer 3
The manuscript by Ardura-Garcia et al presents a longitudinal study on the colonization of E.coli and K. pneumoniae resistant to extended-spectrum lactams over a 6 month period in Cambodian children.
The study is well conducted and the little drop in follow-up is a very positive aspect of this study.

We thank the reviewer for the positive feedback.

Major comments
The authors provided a very comprehensive limitations section which is very good. However, one of the limitations not currently state herein is the confirmation of β-lactamase status in the methods section. Authors talks about ESBL-producing, but as the ESBL is not confirmed by laboratory methods, it would be better to express the results in terms of “extended-spectrum cephalosporin resistant”, resistant to third-generation cephalosporins, or something similar. This would be more precise in line with the methods authors have used and can be confirmed in the antibiotic susceptibility testing table provided. It is true that these isolates are more likely to be ESBL-producers than being resistant by other mechanisms, but authors also refer to those resistant to carbapenems as “carbapenem-resistant” and not “carbapenemase-producers”. Please correct this throughout the entire manuscript. In addition, one limitation of the

**Reply:** We agree with the reviewer’s suggestion. The isolated bacteria were tested using the Vitek 2 GN84 cards for susceptibility testing, which include detection of ESBL and susceptibility to 3^rd^ generation cephalosporins. We included as ESBL-E those that were either ESBL-positive or resistant to 3^rd^ generation cephalosporins. We have added this detail to the methods section, p.7 lines 171:

*“Antimicrobial susceptibilities were determined by VITEK 2 (bioMerieux) testing using AST-GN84 cards that included ESBL detection, and interpreted using 2022 Clinical and Laboratory Standard Institute (CLSI) Antimicrobial Susceptibility Testing guidelines (22).”*

And p.8 lines 188-190:

*“Outcomes of interest were the presence of ESBL and CRE E. coli and K. pneumoniae in RS, and the time-to-gain or -loss of 3GC-R-Ec and K. pneumoniae colonisation. ‘3GC-R’ was defined as isolates those that were either ESBL-positive or resistant to 3rd generation cephalosporins in the VITEK2 susceptibility testing. A ‘gain’ episode was defined as a visit when a child non-colonised in the previous visit became colonised. A ‘loss’ episode was defined as a visit when a child colonised in the previous visit became non-colonised.”*

We have also modified all the ‘ESBL’ in the manuscript to 3^rd^-generation-resistant (3GC-R).

The introduction would benefit of some re-writing in terms of how the ideas are presented. For example, notice that the first paragraph has many short sentences and this would benefit from linking these ideas of the gastro-intestinal tract AMR colonization with infection a bit better.

**Reply:** Thank you for the suggestion. We have modified the first paragraph of the introduction to increase the flow and readability (introduction, p. 5, lines 98-106):

*“Antimicrobial resistance (AMR) is one of the major current global public health threats and a leading cause of death worldwide (23). The gastrointestinal tract (GI) serves as a key reservoir for potentially pathogenic and AMR bacteria, and colonisation with resistant organisms increases the risk of subsequent AMR infection (24). Although AMR is a worldwide problem, its burden is highest are in low- and middle-income countries (LMICs) (23), with South-East Asia considered the region at greatest risk for emergence and spread of AMR (25). In Cambodia, high colonisation prevalence of extended-spectrum beta-lactamase producing Enterobacterales (ESBL-E) has been documented in children: 55% combined Escherichia coli / Klebsiella pneumoniae in older children presenting to healthcare(1); 92% E. coli and 36% K. pneumoniae in children post-discharge (2).”*

In the discussion, authors mention that indeed no risk factors were found for ESBL colonization in E. coli. Considering that the prevalence of the outcome (third-generation cephalosporin resistance) in E. coli is very high, it might become harder to see effects. This might be achieved with larger sample sizes but this is also harder to achieve with children. With this in mind, authors should potentially find descriptions in the literature about this method on high prevalence outcomes and how this affects Kaplan Meier or Cox regressions. Let’s consider that maybe some of the factors could be influence the time-to-loss, but because the re-colonization is very fast, it would be very difficult to see this in practice. This would be in line with lines  474-476 of the discussion. Please add this in the comments section.

**Reply:** We agree with the reviewer that the baseline colonisation proportion for 3GC-R-Ec was very high, and this reduced the number of potential colonisation ‘gain’ episodes (outcome), which decreases the chances of observing an association with risk factors. However, because of frequent new colonisations (which was the outcome for the longitudinal analysis), during the follow-up there were 160 episodes of colonisation gain and 145 of colonisation loss, a large enough number of events to detect associations in Cox-regression models, if we use the rule of thumb of 10-20 events per variable included in a multivariable model (1).

As for the factors associated with time-to-loss of colonisation, it is also true that we may have missed children who had lost the colonisation but were quickly re-colonised and were therefore recorded as still colonised at the following visit (no gain and no loss). This problem may be overcome when using metagenomics analysis, as re-colonisations with different strains will be correctly identified. This has already been added to the discussion section (p. 18, lines 469-472) following other comments.

One major limitation of the study has to do with strain typing and this is likely linked to the previous point for E. coli. Indeed authors mention this in the limitations section and that is very welcomed. However, one thing not currently discussed is how strain typing could add an additional label of “new-gains” even in persistently colonized individuals. If the colonization status doesn’t change, but the strain changes, this could be seen as a “gain” too. Could authors comment on this?

**Reply:** We do believe this would be the case. We modified slightly the limitation we had already included in the discussion section (discussion section, p.18 lines 465-468):

*“We will anyways perform whole genome sequencing in a future step to study strain-level carriage and to help determine if the isolates identified at two different timepoints in the same child were the same strain or differed, defining gain as the acquisition of a new strain.”*

Still in connection with the results, authors separate the subjects into inpatient and outpatient however this classification is not really used the rest of the manuscript. When looking at characteristics of these individuals in table 1, we see that they are very similar groups, expect for resistant K. pneumoniae and inpatient in the past 3 months. I wonder whether if authors would perform the same analysis but in sub-groups (outpatient vs inpatient) whether some factors would emerge. Could authors comment on this? In addition, can authors provide the adjusted hazard ration for the other covariates (those available as a note for table 2)?

**Reply:** Thank you for your comment. It is true that we did not use this classification for the rest of the manuscript. Following other comments we have received, we now decided to use this table to show the baseline characteristics of the whole cohort and stratified by those who missed all the follow-up visits (lost to follow-up) and those who did not.

As for performing a subgroup analysis for inpatient vs outpatient, we did not plan on doing this for the baseline analysis. However, we did analyse previous healthcare exposure (what the reviewer refers to as ‘inpatient in the past 3 months’) as a risk factor, both ‘any healthcare exposure’ and ‘inpatient exposure’, but this is independent to the child being treated as an outpatient or inpatient at the moment of enrolment to the study. Being an inpatient at enrolment, would appear as an ‘inpatient exposure’ for the subsequent follow-up visit. This exposure was studied as a risk factor for the longitudinal dynamic analysis. As this is the exposure of interest, we did not divide our study sample by this same exposure.

As for the reviewer’s second comment, the other covariates included in the dynamic analysis (Cox-regression), were only used as potential confounders. We did not intend to analyse the impact of gender or age (for example) on the loss or gain of 3GC-R-E. We therefore only show the adjusted hazard ratios of the exposure of interest and not of the potential confounders. We have now included the HR of the potential confounders as Supplementary Table 8. However, we advise that the interpretation of the AHR of the confounders may lead to inappropriate assumptions, what is known as the ‘Table 2 fallacy’ (2).

Minor comments:
-       Line 164: which year is the CLSI guidelines from?

**Reply:** They are the 2022 CLSI guidelines. We have included this detail in the methods (p.7, line 172: “[…] *interpreted using 2022 Clinical and Laboratory Standard Institute (CLSI) Antimicrobial Susceptibility Testing guidelines”*

-       Line 201-2 – in this case it is the legal guardian that provides this, please revise this sentence

**Reply:** We have modified accordingly: “*During the study period, 661 children were eligible and invited to participate, of whom 605 (92%) legal representatives agreed to participate (Figure 1)..”*

-       Line 231-32: please be consistent in reporting percentages for clarity

**Reply:** We are unsure what the reviewer is referring to. These lines (and paragraph) include percentages with no decimal places.

-       Lines 317-19 – do the ranges refer what is observed across all time points?

**Reply:** Yes, that is correct. This is what we mean by ‘at each follow-up visit’: “*For 3GC-Ec, at each follow-up visit, 86-92% of those previously colonised remained colonised and 71-80% of those non-colonised became colonised.”*

-       Line 324 – can authors comment on the “intra-individual temporal variation”? what do would authors want to say more specifically? Potentially this could be developed more in the discussion as this could be the indication that there are several clones present in different time points in persistently colonized individuals.

**Reply:** Thank you for this relevant comment. Indeed, we carried out this analysis as an effort to identify possible strain differences not identified phenotypically. We have added a comment in the discussion, p. 18, lines 467-470:

*“We will anyways perform whole genome sequencing to study strain-level carriage and to help determine if the isolates identified at two different timepoints in the same child were the same strain or differed, defining gain as the acquisition of a new strain. In fact, we did observe an intra-individual temporal variation in the antibiotic susceptibility patterns of the 3GC-R isolates, indicating that it is possible the same child was colonised by different strains at two consecutive timepoints.”*

-       Line 448: please re-write the sentence to be “colonisation is likely related”

**Reply:** We have modified to: “*Most identified risk factors for AMR Enterobacterales colonisation are likely related with gut microbiome health.”*

**References:**

1) Peduzzi, P., Concato, J., Feinstein, A. R., & Holford, T. R. Importance of events per independent variable in proportional hazards regression analysis II. Accuracy and precision of regression estimates. Journal of clinical epidemiology. 1995;48(12), 1503-1510.

2) Westreich D, Greenland S. The table 2 fallacy: presenting and interpreting confounder and modifier coefficients. Am J Epidemiol. 2013;177(4):292-8. doi: 10.1093/aje/kws412.
